# Long-Term Follow-Up of Newborns with 22q11 Deletion Syndrome and Low TRECs

**DOI:** 10.1007/s10875-021-01201-5

**Published:** 2022-01-26

**Authors:** Jenny Lingman Framme, Christina Lundqvist, Anna-Carin Lundell, Pauline A. van Schouwenburg, Andri L. Lemarquis, Karolina Thörn, Susanne Lindgren, Judith Gudmundsdottir, Vanja Lundberg, Sofie Degerman, Rolf H. Zetterström, Stephan Borte, Lennart Hammarström, Esbjörn Telemo, Magnus Hultdin, Mirjam van der Burg, Anders Fasth, Sólveig Oskarsdóttir, Olov Ekwall

**Affiliations:** 1 Department of Pediatrics, Sahlgrenska Academy at University of Gothenburg, Gothenburg, Sweden; 2 Department of Pediatrics, Halland Hospital Halmstad, Halmstad, Region Halland Sweden; 3 Department of Rheumatology and Inflammation Research, Sahlgrenska Academy at University of Gothenburg, Gothenburg, Sweden; 4 Department of Pediatrics, Laboratory for Pediatric Immunology, Willem-Alexander Children’s Hospital, Leiden University Medical Center, Leiden, The Netherlands; 5 Children’s Medical Center, National University Hospital of Iceland, Reykjavík, Iceland; 6 Department of Medical Biosciences, Pathology, Umeå University, Umeå, Sweden; 7 Department of Clinical Microbiology, Umeå University, Umeå, Sweden; 8 Centre for Inherited Metabolic Diseases, Karolinska University Hospital Solna, Stockholm, Sweden; 9 Department of Molecular Medicine and Surgery, Karolinska Institute, Stockholm, Sweden; 10 ImmunoDeficiencyCenter Leipzig (IDCL), Municipal Hospital St. Georg Leipzig, Leipzig, Germany; 11 Department of Biosciences and Nutrition, Neo, Karolinska Institute, Stockholm, Sweden

**Keywords:** TREC, newborn screening, 22q11.2 deletion syndrome, DiGeorge syndrome, severe combined immunodeficiency, T lymphopenia, long-term outcome

## Abstract

**Background:**

Population-based neonatal screening using T-cell receptor excision circles (TRECs) identifies infants with profound T lymphopenia, as seen in cases of severe combined immunodeficiency, and in a subgroup of infants with 22q11 deletion syndrome (22q11DS).

**Purpose:**

To investigate the long-term prognostic value of low levels of TRECs in newborns with 22q11DS.

**Methods:**

Subjects with 22q11DS and low TRECs at birth (22q11Low, *N*=10), matched subjects with 22q11DS and normal TRECs (22q11Normal, *N=*10), and matched healthy controls (HC, *N=*10) were identified. At follow-up (median age 16 years), clinical and immunological characterizations, covering lymphocyte subsets, immunoglobulins, TRECs, T-cell receptor repertoires, and relative telomere length (RTL) measurements were performed.

**Results:**

At follow-up, the 22q11Low group had lower numbers of naïve T-helper cells, naïve T-regulatory cells, naïve cytotoxic T cells, and persistently lower TRECs compared to healthy controls. Receptor repertoires showed skewed V-gene usage for naïve T-helper cells, whereas for naïve cytotoxic T cells, shorter RTL and a trend towards higher clonality were found. Multivariate discriminant analysis revealed a clear distinction between the three groups and a skewing towards Th17 differentiation of T-helper cells, particularly in the 22q11Low individuals. Perturbations of B-cell subsets were found in both the 22q11Low and 22q11Normal group compared to the HC group, with larger proportions of naïve B cells and lower levels of memory B cells, including switched memory B cells.

**Conclusions:**

This long-term follow-up study shows that 22q11Low individuals have persistent immunologic aberrations and increased risk for immune dysregulation, indicating the necessity of lifelong monitoring.

**Clinical Implications:**

This study elucidates the natural history of childhood immune function in newborns with 22q11DS and low TRECs, which may facilitate the development of programs for long-term monitoring and therapeutic choices.

**Supplementary Information:**

The online version contains supplementary material available at 10.1007/s10875-021-01201-5.

## Introduction

Newborn screening for T-cell receptor excision circles (TRECs) enables recognition of infants with severe combined immunodeficiency (SCID) before life-threatening infections occur [1]. While TREC screening was introduced to identify infants with SCID, it also identifies several infants with non-SCID T lymphopenia, for which the natural history and prognostic value of low TRECs are lacking [2]. Follow-up guidelines are missing, as is evidence of benefits from targeted monitoring or treatment.

Most infants with non-SCID syndromic T lymphopenia have 22q11.2 deletion syndrome (22q11DS) [2]. The syndrome presents with a wide clinical spectrum affecting many organ systems [3, 4]. Immunodeficiency with mild to moderate T lymphopenia is seen in approximately 75% of individuals, while hypogammaglobulinemia, impaired vaccine response and predisposition to autoimmune disease have also been described, although less commonly [5–8]. The T lymphopenia seen in 22q11DS is caused by thymic hypoplasia, which varies in severity from the presence of a normal thymus to athymia in less than 1.5% of patients [5, 9]. For these patients, thymus transplantation can enable T-lymphocyte maturation and establishment of protective immunity, although such transplantations are still experimental and not widely available [10, 11]. Hematopoietic stem cell transplantation has also been described as a therapeutic option, but long-term survival is poor since the absence of a thymus makes new T-cell lymphopoiesis impossible and enables only engraftment of post-thymic T cells [12, 13]. The proportions of patients with 22q11DS identified by TREC screening differ between screening programs, due to differences in TREC assay methodology, cut-off values, and follow-up algorithms [14]. Nevertheless, the number of patients with 22q11DS identified in screening programs equals to, or even exceeds, the number of patients with SCID [15, 16]. However, the vast majority of 22q11DS patients identified with low TRECs do not have athymia. Previous reports have indicated that some infants with 22q11DS identified through TREC screening increase their numbers of naïve T lymphocytes over time [2, 17, 18]. It is not known if this increase applies to a larger group of patients with 22q11DS or if it entails long-lasting immunological and clinical normalization. Therefore, the prognostic value of low TRECs in infants with 22q11DS without athymia is unclear. The long-term immune competence of these infants has not been well described and there is no clear consensus as to how to monitor their immune function.

Here, we report a controlled follow-up study of newborns with 22q11DS and low TRECs, with detailed immunologic and clinical assessments performed at a median age of 16 years. These subjects were compared with individuals with 22q11DS and normal levels of TRECs in the neonatal period, as well as with healthy controls. Our primary aim was to investigate if low TRECs in the newborn period were predictive of persistent thymus dysfunction, monitored by TRECs, numbers of naïve T lymphocytes, T-cell receptor repertoires, and T-lymphocyte telomere lengths. In addition, we performed a multivariate factor analysis to investigate the associations of low TRECs with additional immunologic and clinical data available for these patients.

## Methods

A brief description of the methods follows. A complete description is provided in the Online Repository.

### Study Design and Study Population

The original neonatal screening cards for 48 infants with confirmed 22q11DS, born during 1993–2010, and obtained at 2–5 days of age were retrieved from storage and TRECs were analyzed. It has previously been shown that properly stored screening cards can be used for the retrospective analysis of TREC [19]. This cohort of 48 infants and the TREC analysis method have been described previously [14]. From the cohort, 10 consecutive patients with the lowest levels of TRECs at birth (22q11Low), as well as 10 patients with the highest TRECs (22q11Normal), were included in this follow-up study. Since premature birth is associated with low TRECs, individuals born at gestational age below 35 weeks were excluded from the study [20]. For each 22q11Low individual, a healthy control subject matched for age and gender was recruited.

A retrospective review of national health records was performed for all patients with 22q11DS, and all participants filled out a health questionnaire.

The study was approved by the Regional Ethical Review Board at Gothenburg University, Gothenburg, Sweden (Dnr. 520-06) and written informed consent was obtained from subjects and guardians in accordance with the Declaration of Helsinki and its later amendments.

### TREC Analysis and Flow Cytometry at Follow-Up

Fresh whole blood was used to quantify TRECs [21] and to determine lymphocyte numbers and proportions using standard flow cytometry methods [22].

### Sorting of Lymphocyte Subsets and Preparation of DNA

Peripheral blood mononuclear cells were isolated from whole blood samples and subjected to flow cytometry-based sorting of B lymphocytes, naïve and memory helper and cytotoxic T lymphocytes. DNA was extracted from the sorted lymphocyte subsets.

### Analysis of T-Cell Receptor Repertoires

Six replicates of DNA from each sample of naïve T lymphocytes (to allow calculation of clonality) and one replicate from each sample of memory T lymphocytes were amplified, followed by Illumina sequencing of rearranged genes that code for the T-cell receptor β-chains (TRB) [23–25].

### Telomere lengths

Quantitative real-time PCR was used to determine the relative telomere length (RTL) in DNA samples from the sorted lymphocytes [26, 27].

### Cytokines

Plasma levels of IFN-γ, IL-13, IL-17A, IL-21, IL-1β, IL-10, TSLP, and CRP were measured using the commercially available V-PLEX assay (MSD, Rockville, MD).

### Immunoglobulins and Specific Antibodies

Serum samples were subjected to assays for IgG, IgA, IgM, and IgG subclasses, as well as for specific IgG antibodies against *Haemophilus influenzae* type b (Hib), *Streptococcus pneumoniae*, CMV, and EBV.

### FASCIA and ELISPOT

T cell-mediated immune responses were determined using flow cytometric assay for specific cell-mediated immune response in activated whole blood (FASCIA), and enzyme-linked immunospot (ELISPOT) assay was used to assess the immunoglobulin-producing capacities of B lymphocytes in vitro [28, 29].

### Statistical Analysis

The Kruskal-Wallis test followed by Dunn´s test to correct for multiple comparisons was applied for univariate analysis (GraphPad Prism 8 Software; GraphPad Inc., La Jolla, CA), except for the analysis of RTL for which a linear mixed model was used, followed by the Bonferroni correction. A difference showing a *P* value < .05 after correction was regarded as statistically significant.

Multivariate orthogonal projection to latent structures by means of partial least squares discriminant analysis (OPLS-DA, SIMCA software; Sartorius Stedim Data Analytics AB, Umeå, Sweden) was used to screen for differences between classes (22q11Low, 22q11Normal, healthy controls) and included all the available data.

## Results

### Study Population

From the original cohort of 48 patients with 22q11DS, three patients with low TRECs were deceased (Supplementary Table S4), two were lost to follow-up, and one was excluded due to premature birth. One patient with normal TRECs declined to participate and four patients were excluded due to thymectomy during heart surgery.

The clinical characteristics, age at follow-up, and reported health issues of the included subjects are presented in Table 1. The three study groups were similar with respect to gender and age. Heart defects were more common in the 22q11Low group (8 out of 10) than in the 22q11Normal group (6 out of 10), and this was also the case for hypocalcemia, which occurred in 5 out of 10 of the 22q11Low subjects and 3 out of 10 of the 22q11Normal subjects.

**Table 1:** Characteristics of the study participants

**Subject-ID**	**TREC on NBS (copies/μl)**	**Age at follow-up (yrs)**	**Gender**	**Heart defect**	**Thymus appearance at surgery**	**Other malformations and deformities**	**Hypocalcemia**	**Neuro-developmental disorder**	**Significant infections**	**Autoimmunity**	**Allergy and asthma**	**Deviation from vaccination schedule** [30]	**CMV-IgG EBV-IgG**
*22q11Low individuals*													
1	1	20	F	ASD VSD	Non-visible	Cleft palate Umbilical hernia	Prolonged		RSV^#^ Prolonged viral Recurrent OM	Thyrotoxicosis with TSHR-Abs		MMR given at age 10 yrs	CMV+ EBV+
2	2	18	F	IAA VSD	Non-visible		Neonatal	ID	VZV^#^ Prolonged viral *Candida*			No live vaccines given	CMV+ EBV+
3	5	12	M	ASD PA	*	VFI		ADD ID	Prolonged viral		Milk protein allergy		CMV+ EBV+
4	5	23	F	IAA ASD VSD	Non-visible			ID	Prolonged viral Recurrent OM Recurrent pneumonia *Candida*	Hypothyroidism with TPO-Abs			CMV+ EBV+
5	7	8	M	VSD CoA	Non-visible	Polydactyly Inguinal hernia Scoliosis	Neonatal	ADHD ID	Pneumonia Recurrent parotitis Septicemia *Candida*				CMV+ EBV-
6	9	22	F			VFI			RSV^#^	Ulcerative colitis			CMV+ EBV+
7	14	16	M	ToF	Non-visible	Scoliosis		Depression Autism	RSV^#^ Prolonged viral *Candida*				CMV- EBV-
8	15	13	F	IAA ASD VSD	Non-visible	VFI	Neonatal		Pneumonia *Candida*				CMV- EBV-
9	15	16	F	TA VSD	Non-visible	Renal agenesis Scoliosis	Neonatal	ID	Pneumonia Prolonged viral		Pollen allergy Asthma		CMV+ EBV-
10	16	14	M			VFI		ADHD	Prolonged viral Recurrent OM *Candida*		Pollen allergy		CMV- EBV+
*Summary 22q11Low group (N=10)*													
Median	8.1	15.8							4 Significant viral 7 Prolonged viral 3 Recurrent OM 3 Significant bacterial 6 *Candida*				CMV 7+ EBV 6+
Range	1.2–16.0	8.1–22.5											
Number			4 M, 6 F	8			5			3	3		
**Subject-ID**	**TREC on NBS (copies/μl)**	**Age at follow-up (yrs)**	**Gender**	**Heart defect**	**Thymus appearance at surgery**	**Other malformations and deformities**	**Hypocalcemia**	**Neuro-developmental disorders**	**Significant infections**	**Autoimmunity**	**Allergy and asthma**		**CMV-IgG EBV-IgG**
*22q11Normal individuals*													
11	43	23	M			VFI Scoliosis Talipes equinovarus		ADD Autism OCD	RSV^#^ Prolonged viral Pneumonia *Candida*				CMV- EBV-
12	46	15	F	IAA VSD	Non-visible	Scoliosis	Neonatal	ADD ID	Pneumonia				CMV- EBV-
13	54	15	F			VFI		ID ADHD	Recurrent OM				CMV- EBV+
14	54	8	M	VSD PA	Non-visible	Polycystic kidney Undescended testis	Neonatal		VZV^#^ Prolonged viral *Candida*				CMV- EBV+
15	54	23	F	ToF PS	*	VFI Imperforate anus			RSV^#^ Recurrent pneumonia Prolonged viral		Asthma		CMV- EBV+
16	71	17	F			VFI Scoliosis Vertebral anomaly		ID		Coeliac disease	Asthma		CMV- EBV+
17	72	11,5	M	VSD	Non-visible			ID	RSV^#^ Pneumonia		Asthma		CMV- EBV+
18	72	12	F	IAA VSD ASD	Non-visible	Cleft palate Multicystic kidney	Neonatal	ADHD ID	RTI^#^ *Candida*				CMV- EBV+
19	74	23	F			VFI Cleft palate Scoliosis			Pneumonia Viral Meningitis^#^				CMV+ EBV-
20	96	16	F	VSD	Hypoplastic	Clinodactyly		ID	Recurrent OM *Candida*		Asthma	No MMR given	CMV+ EBV+
*Summary 22q11Normal group (N=10)*													
Median	62.5	15.8							6 Significant viral 3 Prolonged viral 2 Recurrent OM 5 Significant bacterial 4 *Candida*				CMV 2+ EBV 7+
Range	43.0–96.0	7.7–23.1											
Number			3 M, 7 F	6			3			1	4		
*Summary Healthy controls (N=10)*													
Median	n.a.	15.5							1 Significant viral 1 Prolonged viral 1 Significant bacterial 1 *Candida*	1 Coeliac disease			CMV 6+ EBV2+
Range		9.2–22.2											
Number			4 M, 6 F								4		

*NBS*, newborn screening; *22q11Low*, individuals with 22q11.2 deletion syndrome and low levels of TRECs upon NBS; *22q11Normal*, individuals with 22q11.2 deletion syndrome and normal levels of TRECs upon NBS; *F*, female; *M*, male; *ASD*, atrial septal defect; *VSD*, ventricular septal defect; *IAA*, interrupted aortic arch; *PA*, pulmonary atresia; *CoA*, aortic coarctation; *ToF*, Tetralogy of Fallot; *TA*, truncus arteriosus; *VFI*, velopharyngeal insufficiency; *ID*, intellectual disability; *ADD*, attention deficit disorder; *ADHD*, attention deficit hyperactivity disorder; *OCD*, obsessive compulsive disorder; *RSV*, respiratory syncytial virus; *OM*, otitis media; *VZV*, varicella zoster virus; *RTI*, respiratory tract infection; *CMV*+, anti-CMV IgG >15 AU/ml; *EBV*+, anti-EBV IgG S/CO >1 to capsid and/or to nuclear antigen. *Data not available. ^#^Requiring hospitalization

### Infections, Autoimmune Disease, Allergy, and Asthma

Significant infections were more frequently reported in both groups of 22q11DS subjects than in the healthy controls, although the frequencies did not differ between the 22q11Low and 22q11Normal groups. Autoimmune diseases were diagnosed in 3 out of 10 of the 22q11Low individuals, compared with 1 out of 10 in the 22q11Normal group and healthy controls, respectively. There were no differences between groups regarding frequencies of allergy or asthma. No adverse reactions to live vaccines were reported (Table 1).

### Primary Outcomes

#### TREC Levels at Follow-Up

**Fig. 1:**
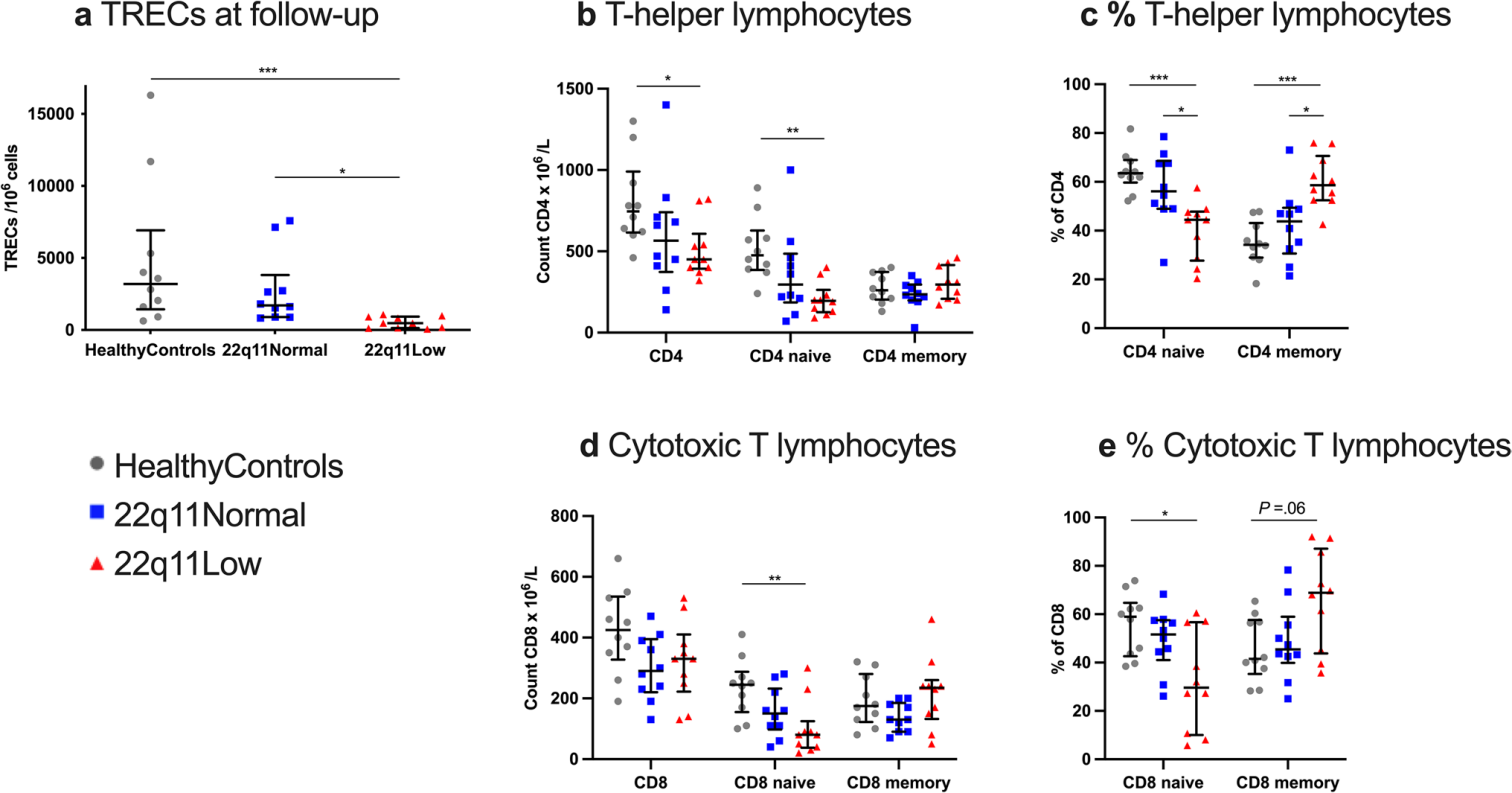
Primary outcome variables at follow-up. **a** The absolute counts of TRECs in whole blood. Absolute counts (**b**) and proportions (**c**) of T-helper lymphocytes (CD4), CD4 naïve cells and CD4 memory cells. Absolute counts (**d**) and proportions (**e**) of cytotoxic T lymphocytes (CD8), CD8 naïve cells and CD8 memory cells. The definitions of the cell types are provided in Supplementary Table S1. Results are shown as individual values for the healthy controls (gray dots), 22q11Normal individuals (blue squares), and 22q11Low individuals (red triangles). Lines denote medians and whiskers indicate the interquartile ranges. *P* value summary indicated on each graph: *P ≤ .05, **P ≤ .01, ***P ≤ .001

In the 22q11Low individuals, the TRECs were lower (477 copies/10^6^ cells), compared with healthy controls (3,195 copies/10^6^ cells) and the 22q11Normal group (1,710 copies/10^6^ cells). No difference in TRECs was noted between the 22q11Normal group and the healthy controls (Fig. 1a and Supplementary Table S5).

#### Naïve T Lymphocytes

The 22q11Low group had lower total numbers of T-helper cells than healthy controls (450 vs. 745×10^6^/L) and the naïve T-helper cells were particularly low (195 vs. 475×10^6^/L), corresponding to a frequency of 44% vs. 63%, respectively, of the T-helper lymphocyte population (Figs. 1b, 1c and Supplementary Table S5). Similarly, the numbers of naïve cytotoxic T cells were lower in the 22q11Low group than in the healthy controls (80 vs. 245×10^6^/L), corresponding to a frequency of 30% vs. 59%, respectively, of all the cytotoxic T lymphocytes (Figs. 1d, 1e and Supplementary Table S5).

Comparing the 22q11Low group with the 22q11Normal group, the numbers of naïve T-helper cells or naïve cytotoxic T cells did not differ, although the proportion of naïve T-helper cells was reduced in the 22q11Low group (44% vs. 56%), (Figs. 1b–1d and Supplementary Table S5).

Naive T cells did not differ between the 22q11Normal group and healthy controls (Figs. 1b–1e).

#### T-Cell Receptor Repertoires

An initial study of the T- and B-cell receptor repertoires using the GeneScan method [31] showed a more-clonal appearance of the TRB repertoires of memory cytotoxic T cells in the 22q11Low individuals compared to the healthy controls (data not shown). To assess the TRB repertoires in more detail, we performed Illumina sequencing of the rearranged V and J genes of the TRB loci of the sorted naïve and memory subpopulations. Only unique sequences (as defined by TRBV gene usage and amino acid sequence of the CDR3) were used for qualitative analysis [24].

**Fig. 2:**
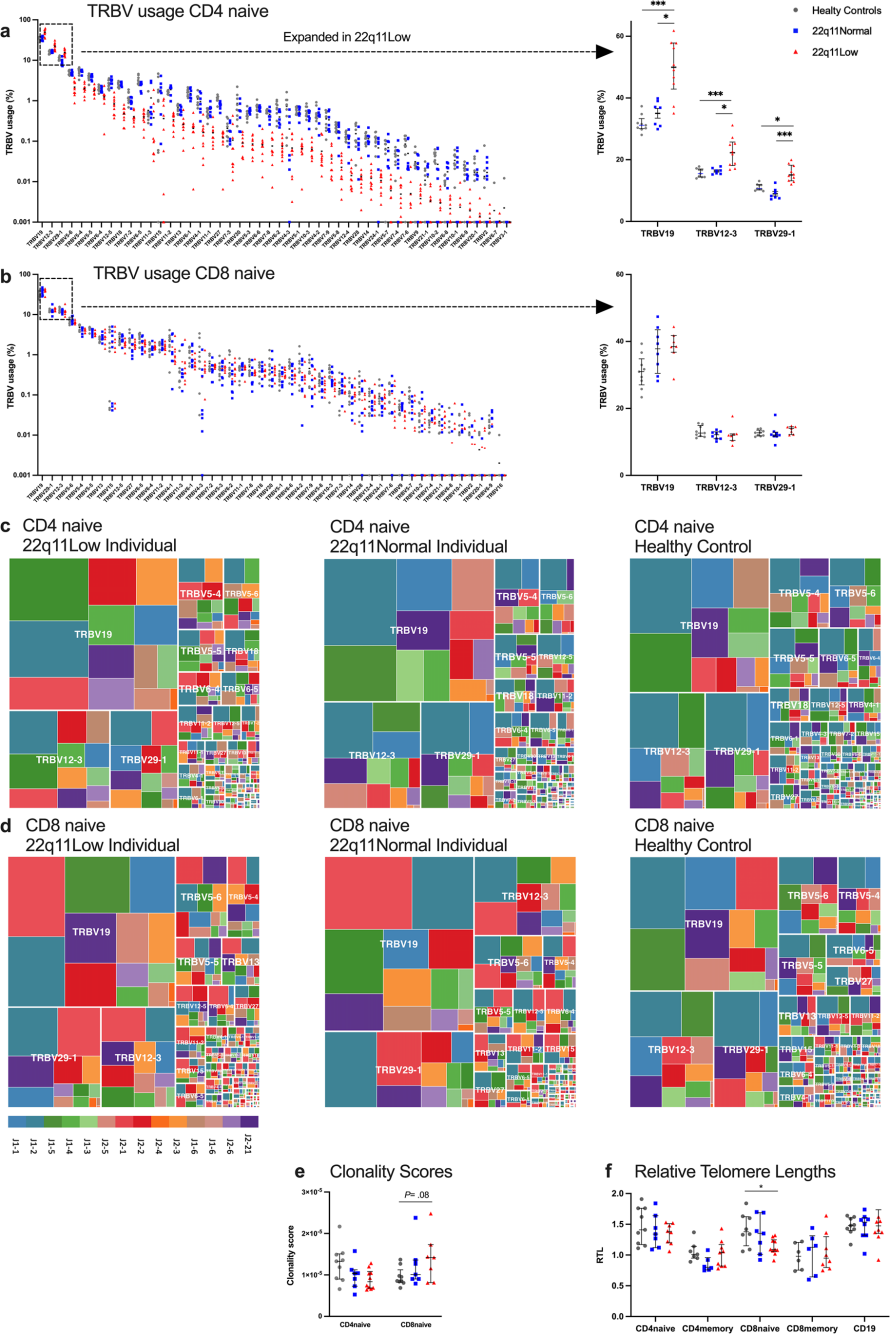
T-cell receptor repertoires and relative telomere lengths. TRBV gene usage in naïve T-helper lymphocytes (CD4 naïve) (**a**) and naïve cytotoxic T lymphocytes (CD8 naïve) (**b**). The TRBV genes are ordered from most-frequently used (left) to least-frequently used (right) in the 22q11Low group. Usage rates of the most frequent TRBV genes are presented in an inset to the far right. Only unique sequences (as defined by TRBV gene usage and amino acid sequence of the CDR3) were used for the T-cell receptor repertoire analysis. Tree plots representing the diversity and clonality of CD4 naïve cells (**c**) and CD8 naïve cells (**d**) in the 22q11Low individuals (left), the healthy control matched for age and gender (right), and the matched 22q11Normal individual (middle). The box size is proportional to the extent of V and J gene usage. Major TRBV genes are indicated on each tree plot, whereas TRBJ genes are identified by the color code at the bottom left. Clonality scores of the CD4 naïve and CD8 naïve cell subsets (**e**). Relative telomere lengths (RTL) in the naïve and memory subsets of CD4, naïve and memory CD8, and B lymphocytes (CD19) (**f**). Results are shown as individual values for the healthy controls (gray dots), 22q11Normal individuals (blue squares), and 22q11Low individuals (red triangles). Information on missing data is provided in Supplementary Table S3. Lines denote medians and whiskers indicate the interquartile ranges. *P* value summary indicated on each graph: *P ≤ .05, **P ≤ .01, ***P ≤ .001

In the 22q11Low individuals, the TRBV gene usage in unique naïve T-helper cells was skewed, with more frequent use of the dominant V genes 19, 12-3 and 29-1 and a lower representation of remaining V genes (Figs. 2a and 2c). Two TRBJ genes were used less frequently in the naïve T-helper cells of 22q11Low individuals compared to the other groups (Supplementary Fig. S3c). The TRBV and TRBJ gene usage in naïve cytotoxic T cells was comparable between the groups, and this was also the case for the memory helper- and memory cytotoxic T cells (Figs. 2b, 2d and Supplementary Figs. S3a, S3b and S3d–f). No major differences were seen between the groups regarding the TRB junction characteristics or CDR3 lengths (Supplementary Fig. S4).

For the naïve T-helper cells, there were no differences in clonality scores between groups [25]. For the naïve cytotoxic T cells, a trend towards higher clonality scores in the 22q11Low group compared to healthy controls was noted (*P*= .08), (Fig. 2e). These two groups did not differ with respect to history of CMV infection (Table 1).

#### Telomere Lengths

The 22q11Low individuals had shorter RTLs in their naïve cytotoxic T cells, compared with healthy controls (1.09 vs. 1.38). There were no differences between the groups regarding RTLs in the other lymphocyte subpopulations (Fig. 2f).

### Secondary Outcomes, Derived from Multivariate Discriminant Analysis

**Fig. 3:**
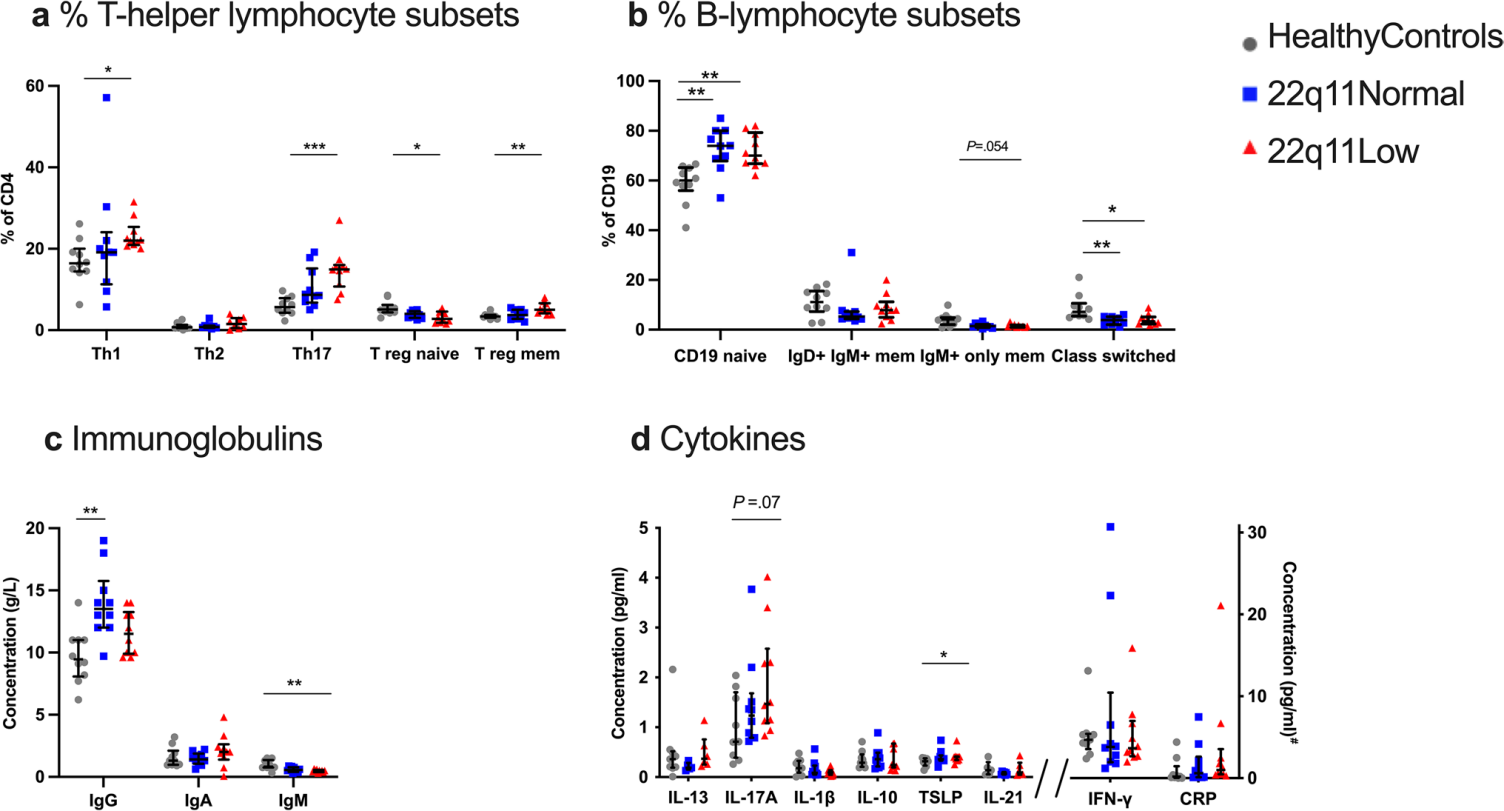
T- and B-lymphocyte subpopulations and immunoglobulin and cytokine levels. **a** Proportions of T-helper lymphocyte subsets (Th1, Th2 and Th17, Treg naïve, Treg memory). **b** Proportions of B-lymphocyte subsets (CD19 naïve, IgD+ IgM+ memory, IgM+ only memory, and class-switched). The definitions of the cell types are provided in Supplementary Table S1. **c** Serum concentrations of IgG, IgA, and IgM. **d** Plasma concentrations of cytokines. ^#^All cytokine concentrations are presented as pg/ml, with the exception of CRP, which is presented in mg/L. IL, Interleukin; TSLP, thymic stromal lymphopoietin; IFN, interferon. Results are shown as individual values for the healthy controls (gray dots), 22q11Normal patients (blue squares), and 22q11Low patients (red triangles). Information on missing data is provided in Supplementary Table S3. Lines denote medians and whiskers indicate the interquartile ranges. *P* value summary indicated on each graph: *P ≤ .05, **P ≤ .01, ***P ≤ .001.

After analysis of the primary outcomes, we performed a multivariate discriminant analysis to investigate the associations of low TRECs on all available data (clinical diagnosis, blood counts, thyroid and parathyroid hormones, lymphocyte populations, TRECs, clonality scores, immunoglobulins, specific antibodies, cytokines, RTL, FASCIA and ELISPOT data). The most important multivariate findings were corroborated by univariate statistical analyses (Fig. 3).

#### Regulatory T Lymphocytes

The 22q11Low group had lower total number of regulatory T cells (Tregs) compared to healthy controls (35 vs. 55×10^6^/L), although the proportions of Tregs did not differ. In the 22q11Low group, there was skewing within the Treg population, with smaller proportions of naïve Tregs (2.8% vs. 5.1%) and larger proportions of memory Tregs, compared with healthy controls (3.7% vs. 2.6%), whereas no differences were seen when the 22q11Low group was compared with the 22q11Normal group (Fig. 3a and Supplementary Table S5).

#### T-Helper Lymphocyte Subsets

When the 22q11Low group was compared to healthy controls, the proportions of Th1 cells were larger (22% vs. 16%) and there was an evident increase in the proportions of Th17 cells (15% vs. 6%), whereas no differences were seen when compared to the 22q11Normal group. Proportions of Th2 lymphocytes did not differ between groups (Fig. 3a and Supplementary Table S5).

#### B Lymphocytes and Immunoglobulin

The 22q11Low group showed skewing within the B-cell compartment compared to healthy controls, with larger proportions of naïve B cells (70% vs. 60%) and smaller proportions of class-switched B cells (3.4% vs. 7.2%). No differences were seen when the 22q11Low group was compared to the 22q11Normal group. However, the 22q11Normal group also differed from healthy controls, having higher percentages of naïve B cells (74% vs. 60%) and lower percentages of class-switched B cells (3.9% vs. 7.2%), (Fig. 3b and Supplementary Table S5).

Immunoglobulin M levels were lower in the 22q11Low group than in healthy controls (0.5 vs. 0.8 g/L), whereas there were no differences of IgG or IgA levels. There were no differences in any of the isotypes between the 22q11Low group and the 22q11Normal group (Fig. 3c).

#### Cytokines

We further evaluated if the skewing of lymphocyte subsets was reflected in the cytokine profiles in plasma. The concentration of TSLP was higher in the 22q11Low group than in the healthy controls (0.41 vs. 0.32 pg/mL), whereas for IL-17A, the interleukin produced by Th17 cells, there was a trend towards higher concentrations in the 22q11Low group, compared with the healthy controls (1.47 vs. 0.71 pg/mL, *P*= .07). The concentrations of IFN-γ, IL-13, IL-21, IL-10, and IL-1β did not differ between groups (Fig. 3d).

#### Functional Tests of T and B Cells

There were no differences between groups with respect to T lymphocyte proliferative response after stimulation with PHA, PWM, PPD, Tetanus toxoid, *Candida*, Influenza A, CMV, EBV, varicella zoster virus and herpes simplex type 1 virus *in vitro*, or with respect to B lymphocyte immunoglobulin production after EBV or PWM stimulation *in vitro *or regarding levels of specific antibodies to Hib, *Streptococcus pneumoniae* or tetanus toxoid (data not shown).

### Multivariate Discriminant Analysis

**Fig. 4:**
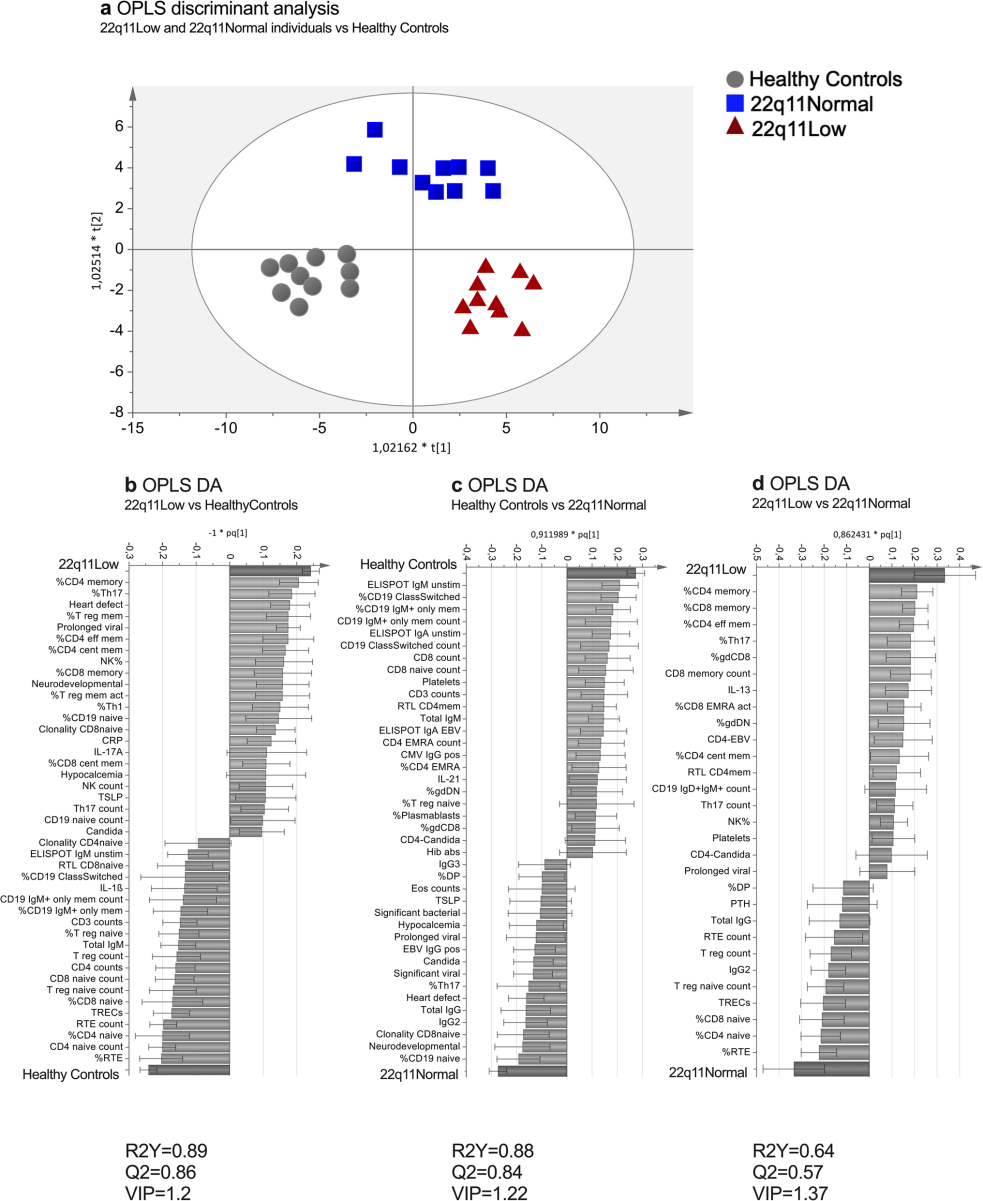
Multivariate discriminant analysis (DA) based on the immunological data and clinical data at follow-up. **a** OPLS-DA score plot showing healthy controls (gray dots), 22q11Normal (blue squares), and 22q11Low (red triangles) individuals, respectively. OPLS-DA column loading plots depicting the associations between the *X*-variables and the following groups: 22q11Low and healthy controls (b); 22q11Normal and healthy controls (**c**); and 22q11Low and 22q11Normal (**d**). The *X*-variables represented by a bar (light grey) pointing in the same direction as a group (dark grey bar) are positively associated with that group, whereas variables pointing in the opposite direction are inversely related. The larger the bar and the smaller the error bar, the stronger is that variable´s contribution to the model. All variables included in the model are listed in Supplementary Table S3. Only the *X*-variables with the strongest contributions to the model are shown and variable influence on projection (VIP) thresholds are indicated.

A score plot derived from the OPLS-DA showed a clear separation between the three study groups (Fig. 4a). Variables that contributed the most to differences between groups are shown in OPLS-DA column plots (Fig. 4b–d). The 22q11Low group was associated with large proportions of memory T cells and small proportions of naïve T cells and TRECs. Interestingly, large proportions of Th17 lymphocytes showed a strong association with the 22q11Low group. Compared to healthy controls, both the 22q11Low and 22q11Normal groups were associated with large proportions of naïve B cells, whereas there was a negative association with memory B cells (Figs. 4b*,* 4c). In addition, the 22q11Normal group was associated with a high concentration of IgG (Fig. 4c). The OPLS-DA for the 22q11Low subjects and the healthy controls generated a good and predictive model (*R*2*Y*=0.89, *Q*2=0.86), which was also the case for the model including 22q11Normal subjects and healthy controls (*R*2*Y*=0.88, *Q*2=0.84). The model including 22q11Low subjects and 22q11Normal subjects was less predictive (*R*2*Y*=0.64, *Q*2=0.57), (Fig. 4b–d).

## Discussion

In this study, we show that low levels of TRECs during the newborn period for infants with 22q11DS are predictive of a long-term impairment of thymic output with defects in the T-cell compartments, bias in TRBV usage and signs of premature ageing of naïve cytotoxic T cells.

T lymphopenia in 22q11DS has been described as a continuum with varying degrees of severity, from near-normal T-cell populations to a complete absence of T cells [3]. Although most infants with 22q11DS identified by TREC screening will not have athymia, we suggest that those identified represent the more-severe end of the immunological spectrum.

Given the low thymic output and low numbers of naïve T cells, we hypothesized alterations in the naïve T-cell receptor repertoires in the 22q11Low individuals, and indeed we found a clear skewing of TRBV gene usage in their naïve T-helper cells. We speculate that the overrepresented V-genes encode receptors with high affinities for self-peptide–MHC complexes, favoring survival during positive selection in the thymus or possibly during the peripheral homeostatic proliferation of naïve T cells [32].

Despite these qualitative alterations of naïve TCR repertoires, we found that the numbers of unique TCR clones of naïve T-helper cells in the 22q11Low individuals were comparable to those in the healthy controls. Previous reports based on flow cytometry or spectratyping have demonstrated a greater perturbation of the receptor repertoires of T-helper cells in individuals with 22q11DS. However, those studies were based on analyses of mixed naïve and memory T-helper cell subsets, which means that they reflected not only thymic output, but probably also to a large degree the infectious history of the individual [33–36].

Our findings of shorter telomere lengths and a trend towards higher clonality scores in naïve cytotoxic T lymphocytes in 22q11Low individuals suggest a history of more-frequent replication in this cell subset. This mimics the effects of human ageing on the cytotoxic T lymphocyte population, whereby naïve cytotoxic T cells are affected earlier, proliferate more rapidly, and develop a higher degree of clonality compared to naïve T-helper cells [37]. For the T-helper subsets, the telomere lengths and clonality scores were not affected. In a previous study of adults with 22q11DS, lower levels of TRECs and shorter telomere lengths in the naïve T-helper cells were found and interpreted as signs of homeostatic proliferation [34]. The younger age of our study participants may explain why this phenomenon was not observed in our study.

We speculate that the TRBV gene usage bias could be a consequence of disturbed thymic selection, whereas the differences in telomere lengths and clonality scores reflect the consequences of peripheral homeostatic mechanisms within the T-cell compartment.

Our finding of persistent T lymphopenia in 22q11Low contrasts with a previous report on the Californian TREC screening, where they found that these infants reached normal numbers of T cells during a shorter follow-up period [2]. Comparisons with that study are, however, problematic because it did not include specific information on the 22q11DS infants, e.g., regarding the lymphocyte subpopulations and duration of follow-up.

Despite the persistent T lymphopenia in 22q11Low individuals, they did not suffer from opportunistic infections, and live vaccines were administered to most and were well tolerated. The history of infections was comparable between the 22q11Low and 22q11Normal groups, in line with previous studies indicating that infections in individuals with 22q11DS generally is a consequence of anatomical and gastroenterological anomalies rather than an underlying immunodeficiency [38]. Of note is that three patients with low TRECs were excluded from the follow-up, since they deceased in infancy. One of them died from a CMV infection, and this patient had undetectable TREC, absence of T cells and would have benefited from early identification by newborn screening [14]. The deaths of the other two infants were related to their cardiac defects.

We contend that the persistent naïve T lymphopenia seen in 22q11Low individuals warrants attention and medical follow-up, as T lymphopenia is known from previous studies to entail an increased risk of developing autoimmune disease [38–41]. Autoimmune disease is commonly associated with 22q11DS, and previous studies have shown that individuals with 22q11DS and autoimmunity have lower levels of T-helper cells and reduced proportions of naïve T-helper cells [8, 38–41]. Similar immunologic alterations, and increased risks for autoimmune disease, have been found following thymectomy performed during early cardiac surgery [42, 43]. The clinical consequences of T lymphopenia in 22q11Low individuals might not become evident until later in life, thereby warranting long-term monitoring.

We found an increased proportion of Th17 lymphocytes in the 22q11Low individuals, which could be secondary to infections with specific microbes, such as *Candida* species [44]. Th17 lymphocytes are known to contribute to the pathogenesis of various autoimmune diseases, which 22q11DS individuals are at higher risk of [8, 38–41, 44]. Our findings are supported by a previous study that showed increased Th17 proportions in adults with 22q11DS and reported a correlation with increased risk of psychosis for 22q11DS individuals [45]. Further studies are needed to confirm our findings and to study the role of Th17 cells in the disease manifestations of 22q11DS.

The present study reveals impaired maturation of peripheral blood B-lymphocytes in both groups of 22q11DS individuals. The skewing of B-lymphocyte subpopulations did not correlate with the TREC levels at newborn screening or at follow-up, in-line with previous studies that could not show that the B-cell defect in 22q11DS individuals is T-cell dependent [46, 47]. Despite the disturbed maturation of B lymphocytes, none of the 22q11DS subjects had hypogammaglobulinemia; instead, they had high IgG levels, which could be due to the chronic burden of infections or dysregulated immune responses. The aberrations in the B-lymphocyte compartment should be studied further, since together with T lymphopenia, they might contribute to the predisposition to autoimmune disease seen in patients with 22q11DS [8, 38–41]. Although T cells depend on a well-functioning thymus for their maturation and selection, there is also a population of B cells in the thymus, which have been described as differing from circulating B cells and are suggested to be of importance for central tolerance induction [48, 49]. Possibly, the peripheral B-cell aberrations in 22q11DS reflect a defect in the thymic B-cell compartment.

The TREC levels in the 22q11Low group were in the range of 1–16 copies/μl. However, differences in methodology between screening programs make it difficult to extrapolate these numbers to other settings. It has previously been shown that the cut-off level for TRECs has an impact on the proportion of infants with 22q11DS who are identified in screening programs [14, 50, 51]. Some screening programs have adjusted their cut-off values for TRECs downwards, in order to decrease the recall rate and increase the specificity for SCID [52]. We propose that infants with 22q11DS identified in screening programs will be fewer in number and more-severely affected by T lymphopenia, such that they will benefit even more from follow-up protocols.

The main strength of this study is its extensive immunological phenotyping and the main limitation is the low number of participants. The trends observed comparing the 22q11Low and 22q11Normal groups might become statistically significant with larger sample sizes. Significant value for the study was created by the long-term follow-up made possible by the analysis of TRECs on the original newborn screening cards. Even so, the study participants were young at follow-up and we suspect that the immunologic abnormalities in the 22q11Low individuals will worsen with time. This highlights the need for continuous follow-up beyond childhood.

## Conclusions

This long-term follow-up study shows that individuals with 22q11DS and low TRECs, who are identifiable in neonatal screening programs, have persistent immunologic aberrations, translating into increased risks for immune dysregulation/autoimmune diseases, and requiring long-term monitoring.

## Supplementary Information

Below is the link to the electronic supplementary material.

Supplementary file1 (PDF 11326 kb)

## Data Availability

The datasets generated during and analyzed during the current study are available from the corresponding author on reasonable request.

## Abbreviations

22q11DS: 22q11.2 deletion syndrome
CDR3: Complementarity determining region
CMV: Cytomegalovirus
CRP: C-reactive protein
EBV: Epstein-Barr virus
IFN: Interferon
IL: Interleukin
Ig: Immunoglobulin
OPLS-DA: Orthogonal projection to latent structures by means of partial least squares discriminant analysis
PHA: Phytohemagglutinin
PWM: Pokeweed mitogen
RTL: Relative telomere length
SCID: Severe combined immunodeficiency
TRB: T-cell receptor β-chain
TREC: T-cell receptor excision circle
Th17: T helper type 17 cell
Treg: Regulatory T cell
TSLP: Thymic stromal lymphopoietin

## Code Availability

Treemap plots of VDJ diversity were generated using R base graphics treemap and RColorBrewer (https://cran.r-project.org/web/packages/treemap/index.html; http://cran.r-project.org/package=RColorBrewer).

## References

[1] Puck JM (2012). Laboratory technology for population-based screening for severe combined immunodeficiency in neonates: the winner is T-cell receptor excision circles. J Allergy Clin Immunol.

[2] Amatuni GS, Currier RJ, Church JA, Bishop T, Grimbacher E, Nguyen AA (2019). Newborn screening for severe combined immunodeficiency and T-cell lymphopenia in California, 2010-2017. Pediatrics.

[3] Maggadottir SM, Sullivan KE (2013). The diverse clinical features of chromosome 22q11.2 deletion syndrome (DiGeorge syndrome). J Allergy Clin Immunol Pract.

[4] McDonald-McGinn DM, Sullivan KE, Marino B, Philip N, Swillen A (2015). 22q11.2 deletion syndrome. Nat Rev Dis Primers.

[5] Morsheimer M, Brown Whitehorn TF, Heimall J, Sullivan KE (2017). The immune deficiency of chromosome 22q11.2 deletion syndrome. Am J Med Genet A.

[6] Gennery AR, Barge D, O´Sullivan JJ, Flood TJ, Abinum M and Cant AJ. (2002). Antibody deficiency and autoimmunity in 22q11.2 deletion syndrome. Arch Dis Child.

[7] Finocchi A, Di Cesare S, Romiti ML, Capponi C, Rossi P, Carsetti R (2006). Humoral immune responses and CD27+ B cells in children with DiGeorge syndrome (22q11.2 deletion syndrome). Pediatr Allergy Immunol.

[8] Tison B, Nicholas SK, Abramson SL, Hanson IC, Paul ME, Seeborg FO (2011). Autoimmunity in a cohort of 130 pediatric patients with partial DiGeorge syndrome. J Allergy Clin Immunol.

[9] Ryan AK, Goodship JA, Wilson DI, Philip N, Levy A, Seidel H (1997). Spectrum of clinical features associated with interstitial chromosome 22q11 deletions: a European collaborative study. J Med Genet.

[10] Davies EG, Cheung M, Gilmour K, Maimaris J, Curry J, Furmanski A (2017). Thymus transplantation for complete DiGeorge syndrome: European experience. J Allergy Clin Immunol.

[11] Markert ML, Devlin BH, Alexieff MJ, Li J, McCarthy EA, Gupton SE (2007). Review of 54 patients with complete DiGeorge anomaly enrolled in protocols for thymus transplantation: outcome of 44 consecutive transplants. Blood..

[12] McGhee SA, Garcia Lloret M, Stiehm R (2009). Immunologic reconstitution in 22q deletion (DiGeorge) syndrome. Immunol Res.

[13] Janda A, Sedlacek P, Hönig M, Friedrich W, Champagne M, Matsumoto T (2010). Multicenter survey on the outcome of transplantation of hematopoietic cells in patients with the complete form of DiGeorge anomaly. Blood..

[14] Lingman Framme J, Borte S, von Döbeln U, Hammarström L, Óskarsdóttir S (2014). Retrospective analysis of TREC based newborn screening results and clinical phenotypes in infants with the 22q11 deletion syndrome. J Clin Immunol.

[15] Kwan A, Abraham RS, Currier R, Brower A, Andruszewski K, Abbott JK (2014). Newborn screening for severe combined immunodeficiency in 11 screening programs in the United States. JAMA.

[16] Argudo-Ramirez A, Martín-Nalda A, Marín-Soria JL, López-Galera RM, Pajares-García S, González de Aledo-Castillo JM (2019). First universal newborn screening program for severe combined immunodeficiency in Europe. Two-years’ experience in Catalonia (Spain). Front Immunol.

[17] Knutsen AP, Baker MW, Markert ML (2011). Interpreting low T-cell receptor excision circles in newborns with DiGeorge anomaly: importance of assessing naive T-cell markers. J Allergy Clin Immunol.

[18] Barry J, Crowley TB, Jyonouchi S, Heimall J, Zackai E, Sullivan K (2017). Identification of 22q11.2 deletion syndrome via newborn screening for severe combined immunodeficiency. J Clin Immunol.

[19] Borte S, von Döbeln U, Fasth A, Wang N, Janzi M, Winiarski J (2012). Neonatal screening for severe primary immunodeficiency diseases using high-throughput triplex real-time PCR. Blood..

[20] Göngrich C, Ekwall O, Sundin M, Brodszki N, Fasth A, Marits P (2021). First year of TREC-based national SCID screening in Sweden. Int J Neonatal Screen.

[21] van Zelm MC, van der Burg M, Langerak AW, van Dongen JJM (2011). PID comes full circle: applications of V(D)J recombination excision circles in research, diagnostics and newborn screening of primary immunodeficiency disorders. Front Immunol.

[22] Maecker HT, McCoy JP, Nussenblat R (2012). Standardizing immunophenotyping for the Human Immunology Project. Nat Rev Immunol.

[23] Gudmundsdottir J, Lundqvist C, IJspeert H, van der Slik E, Óskarsdóttir S, Lindgren S (2017). T-cell receptor sequencing reveals decreased diversity 18 years after early thymectomy. J Allergy Clin Immunol.

[24] IJspeert H, van Schouwenburg P, van Zessen D, Pico-Knijnenburg I, van der Burg M. (2017). Antigen receptor galaxy: a user-friendly, web-based tool for analysis and visualization of T and B cell receptor repertoire data. J Immunol.

[25] Boyd SD, Marshall EL, Merker JD, Maniar JM, Zhang LN, Sahaf B (2009). Measurement and clinical monitoring of human lymphocyte clonality by massively parallel VDJ pyrosequencing. Sci Transl Med.

[26] Cawthon RM (2002). Telomere measurement by quantitative PCR. Nucleic Acids Res.

[27] Henckel E, Landfors M, Haider Z, Kosma P, Hultdin M, Degerman S (2020). Hematopoietic cellular aging is not accelerated during the first 2 years of life in children born preterm. Pediatr Res.

[28] Marits P, Wikström AC, Popadic D, Winqvist O, Thunberg S (2014). Evaluation of T and B lymphocyte function in clinical practice using a flow cytometry based proliferation assay. Clin Immunol.

[29] Czerkinsky CC, Nilsson LÅ, Nygren H, Ouchterlony Ö, Tarkowski A (1983). A solid-phase enzyme-linked immunospot (ELISPOT) assay for enumeration of specific antibody-secreting cells. J Immunol Methods.

[30] Vaccination program for children –Public health agency of Sweden. Available at https://www.folkhalsomyndigheten.se/the-public-health-agency-of-sweden/communicable-disease-control/vaccinations/vaccination-programmes/. Accessed 6 Sept 2021.

[31] van Dongen JJ, Langerak AW, Bruggemann M, Evans PA, Hummel M, Lavender FL (2003). Design and standardization of PCR primers and protocols for detection of clonal immunoglobulin and T-cell receptor gene recombinations in suspect lymphoproliferations: report of the BIOMED-2 Concerted Action BMH4-CT98-3936. Leukemia..

[32] Surh CD, Sprent J (2008). Homeostasis of naive and memory T cells. Immunity..

[33] McLean-Tooke A, Barge D, Spickett GP, Gennery AR (2011). Flow cytometric analysis of TCR Vbeta repertoire in patients with 22q11.2 deletion syndrome. Scand J Immunol.

[34] Piliero L, Sanford A, McDonald-McGinn D, Zackai E, Sullivan K (2004). T-cell homeostasis in humans with thymic hypoplasia due to chromosome 22q11.2 deletion syndrome. Blood..

[35] Nikolich-Zugich J (2008). Ageing and life-long maintenance of T-cell subsets in the face of latent persistent infections. Nat Rev Immunol.

[36] Krishna C, Chowell D, Gönen M, Elhanati Y, Chan TA (2020). Genetic and environmental determinants of human TCR repertoire diversity. Immun Ageing.

[37] Goronzy J, Fang F, Cavanagh M, Qi Q, Weyand C (2015). Naive T cell maintenance in human ageing. J Immunol.

[38] Giardino G, Radwan N, Koletsi P, Morrogh DM, Adams S, Ip W (2019). Clinical and immunological features in a cohort of patients with partial DiGeorge syndrome followed at a single center. Blood..

[39] Montin D, Marolda A, Licciardi F, Robasto F, Di Cesare S, Ricotti E (2019). Immunophenotype anomalies predict the development of autoimmune cytopenia in 22q11.2 deletion syndrome. J Allergy Clin Immunol Pract.

[40] Deshpande DR, Demirdag YY, Marsh RA, Sullivan KE, Orange JS, USIDNET Consortium (2021). Relationship between severity of T cell lymphopenia and immune dysregulation in patients with DiGeorge syndrome (22q11.2 deletions and/or related TBX1 mutations): a USIDNET study. J Clin Immunol.

[41] Crowley TB, Campbell IM, Liebling EJ, Lambert MP, Levitt Katz LE, Heimall J (2021). Distinct immune trajectories in patients with chromosome 22q11.2 deletion syndrome and immune-mediated diseases. J Allergy Clin Immunol.

[42] Gudmundsdottir J, Óskarsdóttir S, Skogberg G, Lindgren S, Lundberg V, Berglund M (2016). Early thymectomy leads to premature immunologic ageing: an 18-year follow-up. J Allergy Clin Immunol.

[43] Gudmundsdottir J, Söderling J, Berggren H, Óskarsdóttir S, Neovius M, Stephansson O (2018). Long-term clinical effects of early thymectomy: associations with autoimmune diseases, cancer, infections, and atopic diseases. J Allergy Clin Immunol.

[44] Bedoya SK, Lam B, Lau K, Larkin J (2013). Th17 cells in immunity and autoimmunity. Clin Dev Immunol.

[45] Vergaelen E, Schiweck C, Van Steeland K, Counotte J, Veling W, Swillen A (2018). A pilot study of immunopsychiatry in the 22q11.2 deletion syndrome: a role for Th17 cells in psychosis?. Brain Behav Immun.

[46] Derfalvi B, Maurer K, McDonald McGinn D, Zackai E, Meng W, Luning Prak E (2016). B cell development in chromosome 22q11.2 deletion syndrome. Clin Immunol.

[47] Klocperk A, Mejstříková E, Kayserová J, Kalina T, Šedivá A (2015). Low marginal zone like B lymphocytes and natural antibodies characterize skewed B lymphocyte subpopulations in del22q11 DiGeorge patients. Clin Immunol.

[48] Gies V, Guffroy A, Danion F, Billaud P, Keime C, Fauny JD (2017). B cells differentiate in human thymus and express AIRE. J Allergy Clin Immunol.

[49] Lundqvist C, Camponeschi A, Visentini M, Telemo E, Ekwall O, Mårtensson IL (2019). Switched CD21^-/low^ B cells with an antigen-presenting phenotype in the infant thymus. J Allergy Clin Immunol.

[50] Gul KA, Øverland T, Osnes L, Baumbusch L, Pettersen R, Lima K (2015). Neonatal levels of T-cell receptor excision circles (TREC) in patients with 22q11.2 deletion syndrome and later disease features. J Clin Immunol.

[51] Liao HC, Liao CH, Kao SM, Chiang CC, Chen YJ (2019). Detecting 22q11.2 deletion syndrome in newborns with low T cell receptor excision circles from severe combined immunodeficiency screening. J Pediatr.

[52] Vogel BH, Bonagura V, Weinberg GA, Ballow M, Isabelle J, DiAntonio L (2014). Newborn screening for SCID in New York State: experience from the first two years. J Clin Immunol.

