# Supplementary material for: Long-Term Follow-Up of Newborns with 22q11 Deletion Syndrome and Low TRECs
Source: J Clin Immunol. 2022 Jan 26;42(3):618–33. doi: 10.1007/s10875-021-01201-5 (PMC9016018; doi:10.1007/s10875-021-01201-5)
Supplement: Supplementary file 1 — Supplementary file1 (PDF 11326 kb) [file 10875_2021_1201_MOESM1_ESM.pdf]

Supplementary material

Long-term follow-up of newborns with 22q11 deletion syndrome and low TRECs
Journal of Clinical Immunology

Corresponding author:
Jenny Lingman Framme, MD
The Department of Pediatrics, The Sahlgrenska Academy at University of Gothenburg, Gothenburg, Sweden
Jenny.lingman-framme@gu.se

Supplementary Methods

Study population

Inclusion criteria. All patients had 22q11DS confirmed by fluorescence *in situ* hybridization (FISH), multiplex ligation-dependent probe amplification (MLPA) or CGH array. Healthy controls were either related to hospital staff or persons claiming good health who were admitted for minor surgery. *Exclusion criteria* were premature birth (<35 weeks gestational age), thymectomy or symptoms of ongoing infection. Premature birth was used as an exclusion criteria because it is known to affect neonatal TREC numbers [20].

Clinical assessment

A retrospective review of national health records was performed for all patients with 22q11DS and the diagnoses were recorded. All participants filled out a health questionnaire regarding their general health, vaccination status, previous infections, allergies and autoimmune disease.

Assessment of infection severity

Significant viral infections were defined as viral infections that required hospital admission. Viral infections that repeatedly lasted more than 2 weeks were regarded as prolonged. Significant bacterial infections were defined as soft tissue infections or pneumonia (with radiologically proven infiltrates and CRP >50) that occurred more than once or a single episode of septicemia, meningitis or osteomyelitis. Recurrent otitis media was defined as a diagnosis of acute otitis media that required antibiotic treatment more than 5 times per year at any time-point during the follow-up period. Any infection with *Candida* was recorded as a significant infection.

TREC analysis at follow-up

DNA was extracted from fresh, whole blood using the QIAamp Blood Mini Kit (Qiagen, Venlo, The Netherlands), followed by a triplex Real-Time qPCR for TRECs and the endogenous reference gene glyceraldehyde-3-phosphate dehydrogenase (*GAPDH*) [21], performed in the same plate in the LightCycler 480

II instrument (Roche, Basel, Switzerland). The PCR was conducted in a volume of 20 µL, containing 10 µL of LightCycler 480 SYBR Green I Master (Roche), 3 µL of sample DNA at a concentration of 30 ng/µL, 1.4 µL each of the forward and reverse primers, and 4.2 µL dH_2O. The signal joint TREC primer sequences were: forward, 5′-CATCCCTTTCAACCATGCTGACACCTCT-3′ and reverse, 5′-CGTGAGAACGGTGAATGAAGAGCAGACA-3′. The *GAPDH* primer sequences were: forward, 5′-CAGCCCCTTCATACCCTCA-3′ and reverse, 5′-GGACCATATTGAGGGACACA-3′ (Thermo Fisher Scientific, Waltham, MA). Every PCR run included pCR2.1-human TREC and pCR2.1-*GAPDH* gene plasmids (Eurofins, Brussels, Belgium) of known concentrations as standards, as well as human cord blood DNA (30 ng/µL, rich in TRECs) as positive control and dH_2O as negative control. The reaction conditions for all Real-Time PCRs were 10 minutes of Taq hotstart activation at 95°C, followed by 45 cycles of 10 seconds of denaturation at 95°C, 20 seconds of annealing at 66°C and 30 seconds at 72°C, and finally one cycle of melting (95°C for 5 seconds, 65°C for 1 minute, ramping from 65°C to 97°C with 0.07°C increments/second).
The number of TRECs was calculated according to the following formula: (Mean of TREC quantity/(Mean *GAPDH* quantity/2)) × 10^6 = TREC copies /10^6 leukocytes. The mean quantity of *GAPDH* was divided by 2, to reflect the biallelic nature of this gene.

Flow cytometry

Fresh, whole blood was used to determine the absolute numbers and proportions of T and B lymphocytes and natural killer (NK) cells, as well as the T-helper and T-cytotoxic cell subpopulations, as proposed by the Human Immunophenotyping Consortium with minor modifications [22]. The definitions of cell types, antibodies and dilutions and the flow cytometry gating strategy are provided in Supplemental Tables S1, S2 and Supplemental Fig. S1. Commercially available BD Multitest 6-colour TBNK Reagent and BD Trucount Tubes (BD Biosciences, San Jose, CA) were used according to manufacturer's instructions to enumerate the T and B lymphocytes, NK cells, and T-helper and T-cytotoxic subpopulations. Subsequently, new 100-µl aliquots of whole blood were stained, and the proportions of lymphocytes within the T-helper and T-cytotoxic, B-lymphocyte and T-regulatory subsets were analyzed. Th2 lymphocyte subsets were characterized according to the cell surface expression of chemoattractant receptor-homologous molecules expressed on Th2 cells (CRTH2) using frozen cells as proposed by Cosmi et al. [E1]. The definitions of cell types and information regarding the gating strategy, antibodies and dilutions are provided in Supplemental Tables S1, S2 and Supplemental Fig. S1.

The multicolor analyses were performed on a FACS Canto II flow cytometer and the results were analyzed using the FACSDiva Software ver. 8.0.1 (BD Biosciences). To ensure optimal flow cytometer performance, setup, and reproducibility of the results, CS&T Research Beads (BD Biosciences) were used daily and CD-Chex Plus (Streck, Omaha, NE) was used weekly according to the manufacturers' instructions.

Sorting of lymphocyte subsets and preparation of DNA

Peripheral blood mononuclear cells (PBMCs) were isolated from whole blood by Ficoll-Paque density gradient centrifugation (GE Healthcare Life Sciences, Little Chalfont, UK). Sorting of B lymphocytes, as well as of naïve and memory T-helper cells and cytotoxic T lymphocytes was performed with more than 95% purity using a flow cytometry-based SY3200 cell sorter (Sony Biotechnology Inc., San Jose, CA). The definitions of cell types and information on the gating strategy, antibodies and dilutions are provided in Supplemental Fig. S2, Supplemental Tables S1 and S2. DNA was isolated from each sorted subset using the QIAamp DNA Mini Kit and QIAcube according to manufacturer's instructions (Qiagen).

Analysis of T-cell receptor repertoires

Six replicates of 50 ng of DNA were amplified from each sample of the naïve T-helper cells and naïve cytotoxic T lymphocytes, whereas one replicate of 100 ng of DNA was amplified from each sample of the memory T-helper cells and memory cytotoxic T lymphocytes. Rearrangements of *TRB* genes were amplified using 23 Vβ forward primers and 13 Jβ reverse primers (Sigma-Aldrich Chemie N.V., Zwijndrecht, The Netherlands). Pooled PCR products were purified using the MinElute gel extraction kit (Qiagen) and Agencourt AMPure XP beads (Beckman Coulter, Fullerton, CA). The concentrations of the PCR products were quantified using the Qubit 2.0 fluorometer with the Qubit 1× dsDNA HS Assay Kit (Thermo Fisher Scientific). On the purified PCR pools, adapter ligation was performed with the Kapa Hyper Prep kit (Roche). Thereafter, the libraries were sequenced on the Illumina MiSeq sequencer with the Miseq reagent kit V3 (Illumina, San Diego, CA). Reads were demultiplexed, trimmed and uploaded to the IMGT High V-Quest software and subsequently analyzed with the ARGalaxy tool, as previously described [23, 24]. Information of V and J gene usage, junctional regions, amino acid compositions and lengths of CDR3 regions was extracted. The analysis was based on sequences that were unique to each DNA replicate. Unique was defined as a rearrangement with a specific V gene that generated a unique amino acid sequence of the CDR3 region. The reoccurrence of a certain rearrangement in any of the six replicates was used for the calculation of clonality scores in naïve T-helper cells and naïve cytotoxic T

lymphocytes, as previously described [25]. Treemap plots of VDJ diversity were generated using R base graphics treemap and RColorBrewer (https://cran.r-project.org/web/packages/treemap/index.html; http://cran.r-project.org/package=RColorBrewer).

Cytokines

Unstimulated plasma levels of IFN-g, IL-13, IL-17A, IL-21, IL-1β, IL-10, TSLP and CRP were measured using the commercially available V-PLEX assay and the Cytokine and Proinflammatory Panel 1 kits according to manufacturer's instructions (MSD, Rockville, MD). Plasma samples were diluted 2-fold, except for the analysis of CRP, which was diluted 1:1,000. Data were acquired on the Meso Quickplex SQ 120 reader (MSD).

Telomere length assessment

Relative telomere length (RTL) was determined in DNA extracted from sorted naïve and memory T helper cells and cytotoxic T lymphocytes, as well as B lymphocytes using the quantitative PCR method described by Cawthon, with minor modifications [26, 27].

DNA was analyzed in triplicate wells (384-well optical plate) in separate telomere (TEL) and single-copy gene hemoglobin subunit beta (HBB) reactions on the QuantStudio 6 Flex Real-Time PCR instrument (Applied Biosystems), on two separate occasions. DNA was diluted to a concentration of 1.75 ng/μL in TE buffer (10 mM Tris [pH 7.5], 100 μM EDTA [pH 8.0]) that contained *Escherichia coli* DNA (1.98 ng/μL). Each reaction contained 10.5 ng DNA, 1X QuantiFast SYBR Green PCR Master mix (Qiagen) and either 400 nM TEL1b forward primer/900 nM TEL2b reverse primer or 400 nM HBB3 forward primer/400 nM HBB4 reverse primer in a total volume of 15 μL. (TEL1b, 5´-CGGTTTGTTTGGGTTTGGGTTTGGGTTTGGGTTTGGGTT-3´; TEL2b, 5´- GGCTTGCCTTACCCTTACCCTTACCCTTACCCTTACCCT-3´; HBB3, 5´-TGTGCTGGCCCATCACTTTG-3´; HBB4, 5´- ACCAGCCACCACTTTCTGATAGG-3´). The cycling conditions were 50°C for 1 second, 95°C for 5 minutes, followed by 40 cycles of 95°C for 10 seconds and 56°C for 30 seconds, and ending with a melting curve analysis. TEL and HBB T/S-values were calculated by the $2^{-\Delta Ct}$ method, where ΔCt = CtTEL-CtHBB. The RTL value was generated by dividing the T/S value of the sample with the T/S value of a reference cell line (CCRF-CEM), which was included in all the runs. A standard curve of the reference cell line DNA was included in every run to monitor PCR efficiency.

Immunoglobulins

Levels of IgG, IgA, IgM and IgG subclasses were measured in serum samples, according to the manufacturer's instructions, using the Optilite assay and reagents in the Optilite turbidometric analyzer (Binding Site Group Ltd., Birmingham, UK).

Specific antibodies

Commercially available test kits were used for the measurement of specific IgG antibody levels in serum directed against capsular polysaccharides of *Haemophilus influenzae* type b (Hib), *Streptococcus pneumoniae* (Vacczyme; Binding Site Group) and tetanus toxoid (Euroimmun, Lübeck, Germany), as well as for the detection of IgM and IgG antibodies to CMV and EBV (Abbott Laboratories Inc., Abbott Park, IL).

FASCIA

Flow cytometric assays for specific cell-mediated immune responses in activated whole blood (FASCIA) were performed with slight modifications to the previously described protocol [28]. Fresh, heparinized blood was diluted 1:8 in cell culture medium (RPMI1640; BioWhittaker Inc., Walkersville, MD) with 1% L-glutamine, 1% mercaptoethanol and 0.1 mg/mL gentamicin. Aliquots of diluted blood were mixed 4:1 with either polyclonal mitogen to phytohemagglutinin (PHA) (Thermo Fisher Scientific) or pokeweed mitogen (PWM) (Biochrom Ltd., Cambridge, UK) or specific antigenic stimuli to PPD (Statens Serum Institute, Copenhagen, Denmark), Tetanus toxoid (SBL Vaccine AB, Stockholm, Sweden), *Candida albicans* (Stallergenes Greer, London, UK), influenza A (Sanofi, Paris, France), varicella zoster virus (nucleocapsid), CMV (AD169), herpes simplex type 1 virus (nucleocapsid) (all three from Folkhälsomyndigheten, Stockholm, Sweden) or EBV (in-house). The cells were incubated for 7 days in a humidified atmosphere at 37°C in 5% CO_2 in air. After incubation, the cells were stained, followed by lysis of erythrocytes, washing and analysis in a FACSCalibur flow cytometer (BD Biosciences). Information on the antibodies and dilutions used is provided in Table S2 in the Online Repository. The results are expressed as the numbers of T helper or cytotoxic T lymphoblasts per mL.

ELISPOT

An enzyme-linked immunospot assay was used as previously described to assess the immunoglobulin-producing capacities of B lymphocytes *in vitro* [29]. Fresh PBMCs were stimulated with either the polyclonal T lymphocyte-dependent mitogen PWM (Sigma-Aldrich, St. Louis, MO) or the T lymphocyte-independent mitogen EBV (in-house), followed by incubation for 6 days in cell culture medium (RPMI 1640; Lonza, Basel,

Switzerland) supplemented with 10% heat-inactivated fetal calf serum (Sigma-Aldrich) in a humidified atmosphere at 37°C in CO_2. Control cultures were incubated in medium without stimulants. The cells were then washed, and incubated in four-fold dilutions (stimulated: 10^4, 2×10^3, 4×10^2, 8×10^1 PBMCs per well; unstimulated: 10^5, 2×10^4, 8×10^4, 4×10^3, 8×10^2 PBMCs per well) for 5 hours at 37°C with 5% CO_2 on nitrocellulose plates (Millipore Inc., Burlington, MA) coated overnight with goat anti human IgG (Jackson ImmunoResearch Europe Ltd., Ely, Cambridgeshire, UK) and blocked with 5% fetal calf serum.
Zones of released immunoglobulin around individual cells were visualized by adding biotinylated goat anti-human IgG, IgA or IgM (Sigma-Aldrich), followed by avidin conjugated with alkaline phosphatase (Sigma-Aldrich) and developed with NBT/BCIP Solution (Mabtech AB, Nacka Strand, Sweden). The number of spots in each well, corresponding to the number of antibody-producing cells, was counted in a standardized way. Results are reported as number of spots per 10^6 PBMCs.

Statistical analysis, OPLS-DA

Orthogonal partial least squares discriminant analysis (OPLS-DA) was used to identify and visualize discriminating prospectively collected immunologic and clinical data. A list of the included *X*-variables is provided (Table S3 in the Online Repository). In order to normalize the data, all the *X*-variables were log-transformed. The scale presented on the *y*-axis of the OPLS plot is dimensionless and the loading vector is normalized to unit length. The quality of the OPLS analyses is assessed by the parameters R2Y and Q2, where R2Y indicates how well the variation of variables is explained by the model, and Q2 estimates how well a variable can be predicted by the model (Q2 >0.5 indicates good predictability). The contribution of each *X*-variable to the OPLS model was calculated, and the variables that contributed the most are presented in the final OPLS-DA column loading plots. For each OPLA-DA column loading plot, the variable influence on projection (VIP) values are indicated. VIP values can be used to discriminate between important and unimportant predictors for the model. Univariate statistical analyses were performed to verify the multivariate findings for the *X*-variables that contributed most to the respective multivariate models.

References

E1. Cosmi L, Annunziato F, Iwasaki M, Galli G, Manetti R, Maggi E, et al. CRTH2 is the most reliable marker for the detection of circulating human type 2 Th and type 2 T cytotoxic cells in health and disease. Eur J Immunol. 2000;30:2972-9.

Table S1.

Definition of cell types

**Cell type**	**Abbreviation**	**Cell surface marker**
**T-helper lymphocytes**	CD4	CD3^{+}CD4^{+}
Naïve T-helper lymphocytes*	CD4 naive	CD3^{+}CD4^{+}CCR7^{+}CD45RA^{+}
Recent thymic emigrants	RTE	CD3^{+}CD4^{+}CCR7^{+}CD45RA^{+}CD31^{+}
Memory T-helper lymphocytes[#]	CD4 memory	
Central memory T-helper lymphocytes*	CD4 cent mem	CD3^{+}CD4^{+}CCR7^{+}CD45RA^{-}
Effector memory T-helper lymphocytes*	CD4 eff mem	CD3^{+}CD4^{+}CCR7^{-}CD45RA^{-}
Effector T-helper lymphocytes*	CD4 effector	CD3^{+}CD4^{+}CCR7^{-}CD45RA^{+}
CD45RA^{+} effector memory T-helper lymphocytes	CD4 EMRA	CD3^{+}CD4^{+}CCR7^{-}CD45RA^{low}
Th1	Th1	CD3^{+}CD4^{+}CCR7^{+}CD45RA^{-}<br>CXCR3+CCR6-
Th2	Th2	CD3^{+} CD4^{+}CD8^{-}CD45RA^{-}CRTH2^{+}
Th17	Th17	CD3^{+}CD4^{+}CCR7^{+}CD45RA^{-}CXCR3^{-}CCR6^{+}
**T regulatory lymphocytes**	T reg	CD3^{+}CD4^{+}CCR4^{+}CD25^{hi}CD127^{low}
Naïve T-regulatory lymphocytes*	T reg naive	CD3^{+}CD4^{+}CCR4^{+}CD25^{hi}CD127^{low}CD45RO<br>$^{-}$
Memory T-regulatory lymphocytes*	T reg mem	CD3^{+}CD4^{+}CCR4^{+}CD25^{hi}CD127^{low}CD45RO<br>$^{+}$
**Cytotoxic T lymphocytes**	CD8	CD3^{+}CD8^{+}
Naïve cytotoxic T lymphocytes*	CD8 naive	CD3^{+}CD8^{+}CCR7^{+}CD45RA^{+}
Memory cytotoxic T lymphocytes[#]	CD8 memory	
Central memory cytotoxic T lymphocytes*	CD8 cent mem	CD3^{+}CD8^{+}CCR7^{+}CD45RA^{-}
Effector memory cytotoxic T lymphocytes*	CD8 eff mem	CD3^{+}CD8^{+}CCR7^{-}CD45RA^{-}
Effector cytotoxic T lymphocytes*	CD8 effector	CD3^{+}CD8^{+}CCR7^{-}CD45RA^{+}
CD45RA^{+} effector memory cytotoxic T lymphocytes	CD8 EMRA	CD3^{+}CD8^{+}CCR7^{-}CD45RA^{low}
**Double-positive T lymphocytes**	DP	CD3^{+}CD4^{+}CD8^{+}
**γδ T lymphocytes**	γδ	CD3^{+}TCRαβ^{-}TCRγδ^{+}CD4^{-}CD8^{-}
**B lymphocytes**	CD19	CD3^{-}CD19^{+}
Naïve B lymphocytes	CD19 naive	CD3^{-}CD19^{+}CD20^{+}CD27^{-}<br>24^{low}38^{low}IgD^{+}IgM^{+}
Transitional B lymphocytes	CD19 transitional	CD3^{-}CD19^{+}CD20^{+}CD27^{-}24^{hi}38^{hi}IgD^{+}IgM^{+}
IgD^{+} IgM^{+} memory B lymphocytes	CD19 IgD^{+} IgM^{+} mem	CD3^{-}CD19^{+}CD20^{+}CD27^{+}IgD^{+}IgM^{+}
IgM^{+} only memory B lymphocytes	CD19 IgM^{+} only mem	CD3^{-}CD19^{+}CD20^{+}CD27^{+}IgD^{-}IgM^{+}
Class-switched memory B lymphocytes	CD19 Class switched	CD3^{-}CD19^{+}CD20^{+}CD27^{+}IgD^{-}IgM^{-}
Plasmablasts	Plasmablasts	CD3^{-}CD19^{+}CD20^{-}CD38^{+}CD27^{hi}
**NK cells**	NK	CD3^{-}CD56^{+}16^{+}

*With CD38^{+}DR^{+} as markers of activation (act).
[#]For cell sorting, memory T lymphocytes were defined as CD45RA^{-}, i.e., effector and EMRA subsets were not included.
CD, Cluster of differentiation; CCR, chemokine C receptor; CXCR, chemokine CX receptor; CRTH, chemoattractant receptor homologous molecule expressed on Th2 cells; NK, natural killer.

Table S2.

Antibodies used in the study

**Flow cytometry panel**	**Antibody**[#]	**Clone**	**Dilution factor**
**TBNK** Complete Lymphocyte Subset Panel	6-colour reagent	Catalog no. 644611	5
	CD45-PerCP-Cy 5.5		
	CD3-FITC		
	CD4-PE-Cy7		
	CD8-APC-Cy7		
	CD19-APC		
	CD16/56-PE		
**T lymphocytes**			
	CD3-V450	UCHT1	40
	CD4-PerCP-Cy 5.5	SK3	10
	CD8-APC-H7	SK1	40
	CD45RA-PE-CY7	L48	40
	CD31-FITC	WM59	40
	CCR7-PE	150503	40
	CD38-APC	HB7	40
	HLA-DR-V500	G46-6	40
**T regulatory lymphocytes**			
	CD3-V450	UCHT1	40
	CD4-PerCP-Cy 5.5	SK3	10
	CD45RO-APC-H7	UCHL1	40
	CCR7-FITC	150503	40
	CD25-PE	2A3	10
	CCR4-PE-CY7	1G1	40
	CD127-APC	eBioRDR5	40
	HLA-DR-V500	G46-6	40
**Th1 and Th17 subsets**			
	CD3-V450	UCHT1	40
	CD4-PerCP-Cy 5.5	SK3	10
	CXCR3-PE	1C6/CXCR3	10
	CCR6-PE-CY7	11A9	40
**Th2 and γδ subsets**			
	CRTh2-PE	BM16	20
	CD3-V450	UCHT1	40
	CD4-APC-R700	RPA-T4	20
	CD8-PE-Cy7	RPA-T8	50
	CD45RA-APC-H7	HI100	20
	TCRαβ-BV510	T10B9.1A-31	20
	TCRγδ-PerCP-Cy 5.5	B1	20
**B lymphocytes**			
	CD19-PerCP-Cy 5.5	SJ25C1	10
	CD20-APC-H7	L27	40
	CD27-PE-CY7	M-T271	40
	CD38-APC	HB7	40
	IgM-BB515	G20-127	40
	IgD-V500	IA6-2	40
**FASCIA**[##]			
	CD3-PerCP	SK7	10
	CD4-APC	SK3	50
	CD8-PE	SK1	33
**Sorting of Lymphocytes**			**Added volume antibody (μl /10^6 cells)**
	CD3-V450	UCHT1	2
	CD4-PerCP-Cy5.5	SK3	4
	CD25-PE	2A3	4
	CD127-APC	eBioRDR5	1
	CD45RA-PeCy7	L48	1
	CCR7-FITC	150503	2
	CD8-APC-H7*	SK1	0.5
	CD19-APC-H7[¶]	HIB19	2

[#]All antibodies were from BD Biosciences, with the exception of CD127-APC, which was provided by Thermo Fischer Scientific (Waltham, MA).
[##]FASCIA, flow cytometric assay for specific cell-mediated immune responses in activated whole blood.
[¶]Only used in sorting panel 1.
*Only used in sorting panel 2.

Table S3.

Variables included in the OPLS-DA model

**Clinical diagnosis**	RTE count	CD19 count	**Clonality scores**
Heart defect	%CD4 memory	%CD19 naïve	ClonalityCD4naïve[b]
Autoimmunity	CD4 memory count	CD19 naïve count	ClonalityCD8naïve[c]
Significant viral infections	%CD4 cent mem*	%CD19 transitional	
Prolonged viral infections	CD4 cent mem count*	CD19 transitional count	**Immunoglobulins**
Significant bacterial infections	%CD4 eff mem*	%CD19 IgD+IgM+ mem	Total IgG
Candida	CD4 eff mem count*	CD19 IgD+IgM+ mem count	Total IgA
Asthma/allergy	%CD4 effector	%CD19 IgM+ only mem	Total IgM
Neurodevelopmental	CD4 effector count	CD19 IgM+ only mem count	IgG1
	%CD4 EMRA*	%CD19 Class-switched mem	IgG2
**Blood counts**	CD4 EMRA count	CD19 Class-switched count	IgG3
Hemoglobin	%Th1	%Plasmablasts	IgG4
Leucocytes	Th1 count	Plasmablasts count	
Platelets	%Th2[a]	%NK	**ELISPOT**[d]
Neutrophils	%Th17	NK count	Elispot IgM unstimulated
Eosinophils	Th17 count		Elispot IgG unstimulated
Lymphocytes	%γδCD4[a]	**TRECs**	Elispot IgA unstimulated
Eosinophils	%γδCD8[a]		Elispot IgM EBV
Monocytes	%γδ[a]	**FASCIA**	Elispot IgG EBV
Basophils	%CD8	CD4-unstimulated	Elispot IgA EBV
	CD8 count	CD4-PHA	Elispot IgM PWM
**Thyroid/parathyroid**	%CD8 naïve*	CD4-PWM	Elispot IgG PWM
T4	CD8 naïve count	CD4-PPD	Elispot IgA PWM
TSH	%CD8 memory	CD4-Tetanus toxoid	
Ionized calcium	CD8 memory count	CD4-*Candida*	**Cytokines**[e]
Parathyroid hormone	%CD8 cent mem*	CD4-Influenza A	IFN-γ
	CD8 cent mem count	CD4-CMV	IL-13[f]
**Specific IgG antibodies**	%CD8 eff mem*	CD4-EBV	IL-17A
Tetanus toxoid	CD8 eff mem count*	CD4-VZV	IL-21[g]
Streptococcus pneumoniae	%CD8 effector*	CD4-HSV1	IL-1β[h]
Haemophilus influenzae type B	CD8 effector count*	CD8-unstimulated	IL-10
CMV	%CD8 EMRA*	CD8-PHA	TSLP
EBV	CD8 EMRA count*	CD8-PWM	CRP
	%DP	CD8-PPD	
**Lymphocyte populations**	%T reg	CD8-Tetanus toxoid	**Relative telomere length**
CD3 count	T reg counts	CD8-*Candida*	RTLCD4naïve[i]
%CD4	%T reg naïve	CD8-Influenza A	RTLCD4mem[j]
CD4 count	T reg naïve count	CD8-CMV	RTLCD8naïve[k]
%CD4 naïve*	%T reg mem*	CD8-EBV	RTLCD8mem[l]
CD4 naïve count	T reg mem count*	CD8-VZV	RTLCD19[m]
% RTE	%CD19	CD8-HSV1	

OPLS-DA, Orthogonal projection to latent structures by means of partial least squares discriminant analysis; T4, thyroxine; TSH, thyroid stimulating hormone; CMV, cytomegalovirus; EBV, Epstein-Barr virus; RTE, recent thymic emigrants; FASCIA, flow cytometric assay for specific cell-mediated immune response in activated whole blood; ELISPOT, enzyme-linked immunospot assay; PHA, phytohemagglutinin; PWM, pokeweed mitogen; VZV, varicella zoster virus; HSV1, herpes simplex virus 1; PPD purified protein derivative; TSLP, thymic stromal lymphopoietin; RTL, relative telomere length. Definitions of cell types and abbreviations are provided in Table E1 in the Online Repository. *Corresponding activated subset was included as a separate variable (act).

[a]Data missing from two 22q11Low individuals, two 22q11Normal and one healthy control.
[b]Data missing from three 22q11Normal individuals and one healthy control.
[c]Data missing from three 22q11Low, two 22q11Normal individuals and one healthy control.
[d]Data missing from one 22q11Low individual.
[e]Data missing from one healthy control.
[f]Data from four 22q11Low and four 22q11Normal individuals were below the detection range and excluded.
[g]Data from four 22q11Low, three 22q11Normal individuals and four healthy controls were below the detection range and excluded.
[h]Data from one 22q11Low individual were below detection range and excluded.
[i]Data missing from two 22q11Low, three 22q11Normal individuals and one healthy control.
[j]Data missing from one 22q11Low, four 22q11Normal individuals and three healthy controls.
[k]Data missing from three 22q11Normal individuals and two healthy controls.
[l]Data missing from two 22q11Low, four 22q11Normal individuals and four healthy controls.
[m]Data missing from two 22q11Normal individuals and one healthy control.

Supplemental Table S4.

Characteristics of deceased patients with low TRECs

TREC on NBS (copies/μl)	Age at diagnosis (months)	Age at death (months)	Gender	T lymphocytes ($\times 10^6$/L)* at diagnosis	Heart defect	Thymus appearance at surgery or MRI	Other malformations and deformities	Hypocalcemia	Significant infections	Cause of death
0	3.5	5	F	CD3 0<br>CD4 0<br>CD8 0	Right aortic arch	Non-visible	None	Neonatal	CMV	CMV
1.7	< 1	3.5	M	CD3 510<br>CD4 360<br>CD8 130	TA *type 2*, VSD	Non-visible	Mb Hirschsprung<br>IVH, hydrocephalus<br>VFI	Neonatal		Respiratory insufficiency secondary to aspiration and cardiac failure
8.8	< 1	8	F	CD3 900<br>CD4 650<br>CD8 250	TA *type 3*	Non-visible	Omphalocele<br>Talipes equinovarus	Neonatal	Recurrent fever of unknown origin<br>*Stenotrophomonas maltophilia* in BAL	Bronchospasm and cardiac failure during anesthesia for BAL

NBS, Newborn screening; MRI, magnetic resonance imaging; F, female; M, male; TA, truncus arteriosus; VSD, ventricular septal defect; IVH, intra ventricular hemorrhage; VFI, velopharyngeal insufficiency; CMV, cytomegalovirus; RTI, respiratory tract infection; BAL, bronchoalveolar lavage. * Data not available for naïve T cell counts.

Table S5.

TREC levels and counts and proportions of lymphocyte populations at follow-up

	22q11Low N=10 (Median)	Interquartile Range	22q11Normal N=10 (Median)	Interquartile Range	Healthy Controls N=10 (Median)	Interquartile Range	Adjusted *P*-value 22q11Low vs. HC	Adjusted *P*-value 22q11Low vs. 22q11Normal
TREC (copies/10^6 cells)	477	140–930	1,710	901–3,823	3,195	1,437–6,915	< .001	< .05
Lymphocytes ($\times 10^6$/L)	1500	1,250–1,975	1,350	953–2,000	1,700	1,375–2,225	> .99	> .99
CD3 ($\times 10^6$/L)	835	715–1,150	890	630–1,300	1,350	1,075–1,575	< .05	> .99
%CD3 of lymphocytes	59	50–62	64	61–70	77	71–83	< .001	.22
CD4 ($\times 10^6$/L)	450	392–607	565	372–740	745	615–990	< .05	.31
%CD4 of CD3	56	49–63	58	49–71	59	51–67	> .99	> .99
CD4 naïve ($\times 10^6$/L)	195	125–262	295	185–485	475	385–627	< .001	.31
%CD4 naïve of CD4	44	28–48	56	49–69	63	60–69	< .001	< .05
%CD4 naïve act of CD3	0	0–0	0	0–0.03	0	0–0.03	.53	.66
RTE ($\times 10^6$/L)	125	88–185	240	143–343	350	303–488	< .001	. 27
%RTE of CD4	29	21–36	43	39–55	49	41–52	< .001	< .01
CD4 mem ($\times 10^6$/L)	295	208–415	235	198–295	260	203–373	> .99	.56
%CD4 mem of CD4	59	52–71	44	31–49	35	29–43	< .0001	< .05
CD4 cent mem ($\times 10^6$/L)	160	110–248	160	105–183	160	108–213	> .99	> .99
CD4 cent mem act ($\times 10^6$/L)	0	0–0	0	0–0	0	0–0	.66	> .99
%CD4 cent mem of CD4	34	29–41	23	19–34	21	17–27	< .01	.15
%CD4 cent mem act of CD3	0.2	0.1–0.3	0.2	0.1–0.3	0.1	0–0.2	> .99	> .99
CD4 eff mem ($\times 10^6$/L)	110	75–145	80	75–110	95	58–133	> .99	.59
CD4 eff mem act ($\times 10^6$/L)	0	0–0	0	0–0	0	0–0.01	.59	> .99
%CD4 eff mem of CD4	23	17–29	13	11–22	11	8–18	< .05	.20
%CD4 eff mem act of CD3	0.7	0.4–0.9	0.4	0.3–0.8	0.3	0.2–0.4	.09	> .99
CD4 effector ($\times 10^6$/L)	0	0–0.005	0	0–0	0	0–0.003	> .99	> .99
%CD4 effector of CD4	0	0–0.006	0	0–0	0	0–0.005	> .99	> .99
CD4 EMRA ($\times 10^6$/L)	0	0–0.003	0	0–0	0.01	0–0.02	.07	> .99
%CD4 EMRA of CD4	0	0–0.005	0	0–0	0.02	0–0.03	.01	> .99
%CD4 EMRA act of CD3	0	0–0	0	0–0	0	0–0.1	.27	> .99
T reg ($\times 10^6$/L)	35	18–40	45	38–83	55	40–73	< .05	. 14
%T reg of CD4	6.9	5.5–8.1	7.1	6.0–7.8	7.5	6.7–8.9	. 49	> .99
T reg naïve ($\times 10^6$/L)	15	0–20	30	10–45	35	28–50	< .01	.12
%T reg naïve of CD4	2.8	1.9–4.6	4.0	3.0–4.7	5.1	4.4–6.2	< .05	> .99
T reg mem ($\times 10^6$/L)	20	10–20	20	20–30	20	10–30	> .99	.73
T reg mem act ($\times 10^6$/L)	0	0–10	5	0–10	5	0–10	> .99	> .99
%T reg mem of CD4	3.7	3.3–4.6	3.1	2.3–3.9	2.6	2.2–2.9	< .01	. 40
%T reg mem act of CD3	1.7	1.4–2.5	1.5	0.8–2	0.9	0.7–0.9	< .05	> .99
Th1 ($\times 10^6$/L)	105	80–170	85	80–100	135	98–173	> .99	.72
%Th1 of CD4	22	21–25	19	11–24	16	14–20	< .05	. 14
%Th2 of CD4[a]	1.5	0.5–3.0	0.8	0.6–1.1	0.7	0.5–1.3	. 52	> .99
Th17 ($\times 10^6$/L)	65	48–105	50	40–70	45	30–60	.08	.49
%Th17 of CD4	15	11–16	9	7–15	6	4–8	< .001	. 08
CD8 ($\times 10^6$/L)	330	222–410	290	220–395	425	327–535	.32	> .99
%CD8 of CD3	35	29–40	30	24–36	32	25–40	> .99	.81
CD8 naïve ($\times 10^6$/L)	80	37–125	150	97–232	245	155–287	< .01	.35
%CD8 naïve of CD8	30	10–57	52	41–58	59	43–65	< .05	.35
%CD8 naïve act of CD3	0	0–0	0	0–0.03	0	0–0	> .99	.23
CD8 mem ($\times 10^6$/L)	235	133–260	130	90–185	175	123–280	> .99	.20
%CD8 mem of CD8	69	44–87	45	40–59	42	35–58	.062	.25
CD8 cent mem ($\times 10^6$/L)	35	18–70	20	18–30	20	18–38	.61	.51
%CD8 cent mem of CD8	136	71–166	75	47–130	58	34–84	< .05	.63
%CD8 cent mem act of CD3	0	0–0.2	0	0–0	0	0–0	.95	.87
CD8 eff mem ($\times 10^6$/L)	60	18–95	45	30–68	60	23–128	> .99	> .99
CD8 eff mem act ($\times 10^6$/L)	0	0–0.01	0	0–0.01	0	0–0	> .99	> .99
%CD8 eff mem of CD8	17	10–33	16	10–24	14	6–30	> .99	> .99
%CD8 eff mem act of CD3	0.6	0.3–1.2	0.4	0.16–1	0.4	0.15–0.7	.8	> .99
CD8 effector ($\times 10^6$/L)	20	8–53	20	10–33	35	20–50	.63	> .99
CD8 effector act ($\times 10^6$/L)	0	0–0.1	0	0–0	0	0–0	.23	.23
%CD8 effector of CD8	5	2–17	8	4–11	7	5–13	> .99	> .99
%CD8 effector act of CD3	0.2	0–0.6	0	0–0.2	0.1	0–0.2	> .99	.40
CD8 EMRA ($\times 10^6$/L)	60	28–120	35	20–53	60	40–70	> .99	.52
CD8 EMRA act ($\times 10^6$/L)	0	0–0.1	0	0–0	0	0–0.003	> .99	.51
%CD8 EMRA of CD8	20	9–36	14	8–17	12	11–16	.82	.84
%CD8 EMRA act of CD3	0.4	0–0.7	0.1	0–0.2	0.2	0.1–0.4	> .99	.10
%DP of CD3	0.5	0.3–0.9	0.8	0.5–1.2	0.6	0.3–0.7	> .99	.34
%γδ of CD3[a]	6	3–11	4	3–5	10	4–16	> .99	.49
CD19 ($\times 10^6$/L)	285	195–398	255	120–370	215	160–260	.27	> .99
%CD19 of lymphocytes	19	16–25	19	12–22	12	10–15	< .05	> .99
CD19 naive ($\times 10^6$/L)	215	148–265	185	93–253	125	80–163	< .05	.96
%CD19 naive of CD19	70	67–79	74	68–80	60	56–65	< .01	> .99
CD19 transitional ($\times 10^6$/L)	20	0–73	20	8–33	20	10–23	> .99	> .99
%CD19 transitional of CD19	9	3–15	7	4–8	9	5–11	> .99	> .99
CD19 IgD+IgM+ mem ($\times 10^6$/L)	20	18–33	10	10–20	20	10–30	> .99	.21
%CD19 IgD+IgM+ mem of CD19	7.9	4.9–11.2	5.3	4.4–7.3	11.2	7.3–15.5	> .99	. 76
CD19 IgM+ only mem ($\times 10^6$/L)	0	0–0	0	0–0	10	0–20	< .05	> .99
%CD19 IgM+ only mem of CD19	1.4	1.0–1.9	1.4	0.9–2.2	4.1	2.1–4.9	.058	> .99
CD19 Class Switched mem ($\times 10^6$/L)	10	0–20	5	0–10	20	10–20	.55	.74
%CD19 Class Switched mem of CD19	3.4	2.3–5.1	3.9	2.1–5.2	7.2	5.4–10.6	< .05	> .99
%Plasmablasts of CD19	1.6	0.5–1.8	0.9	0.6–1.6	1.6	1.1–2.4	> .99	> .99
Plasmablasts ($\times 10^6$/L)	0	0–0	0	0–0	0	0–0.003	0.23	> .99
%NK of lymphocytes[b]	19	15–30	17	12–20	9	7–12	< .01	.73
NK cells ($\times 10^6$/L)[b]	284	210–528	213[a]	133–505	157	105–476	< .05	.82

P–value using Kruskal-Wallis test followed by Dunn´s multiple comparisons test. TREC, T–cell receptor excision circles. Definitions of the cell types and abbreviations are provided in Table E1 in the Online Repository. Counts for activated subsets (act) are reported when quantifiable for more than one individual. [a]Missing data from two 22q11Low, two 22q11Normal individuals and one healthy control. [b]Missing data from one 22q11Low.

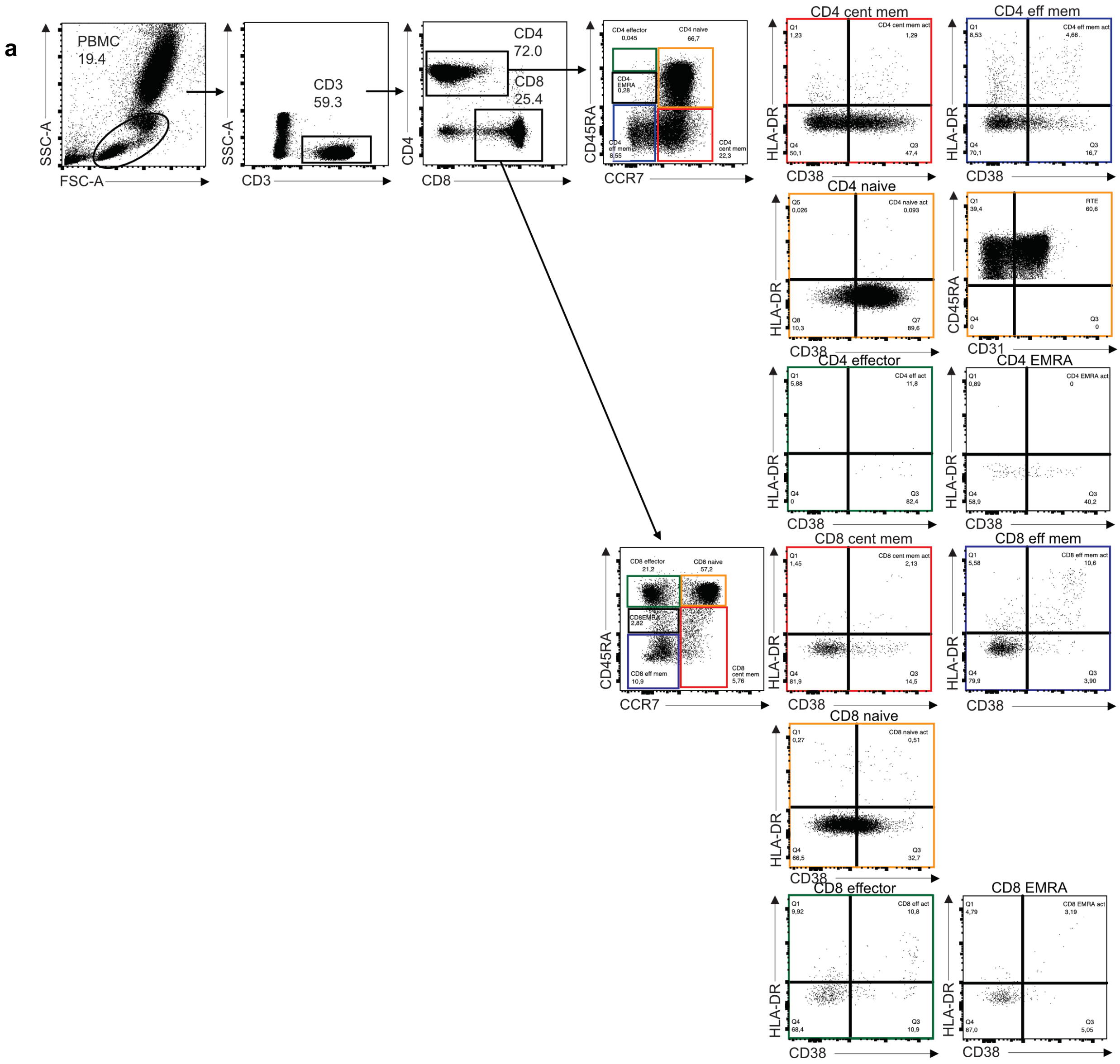

Fig. S1a Gating strategy used for flow cytometry, T-lymphocyte subsets

SSC-A, Side-scatter area; FSC-A, forward-scatter area; FSC-H, forward-scatter height.

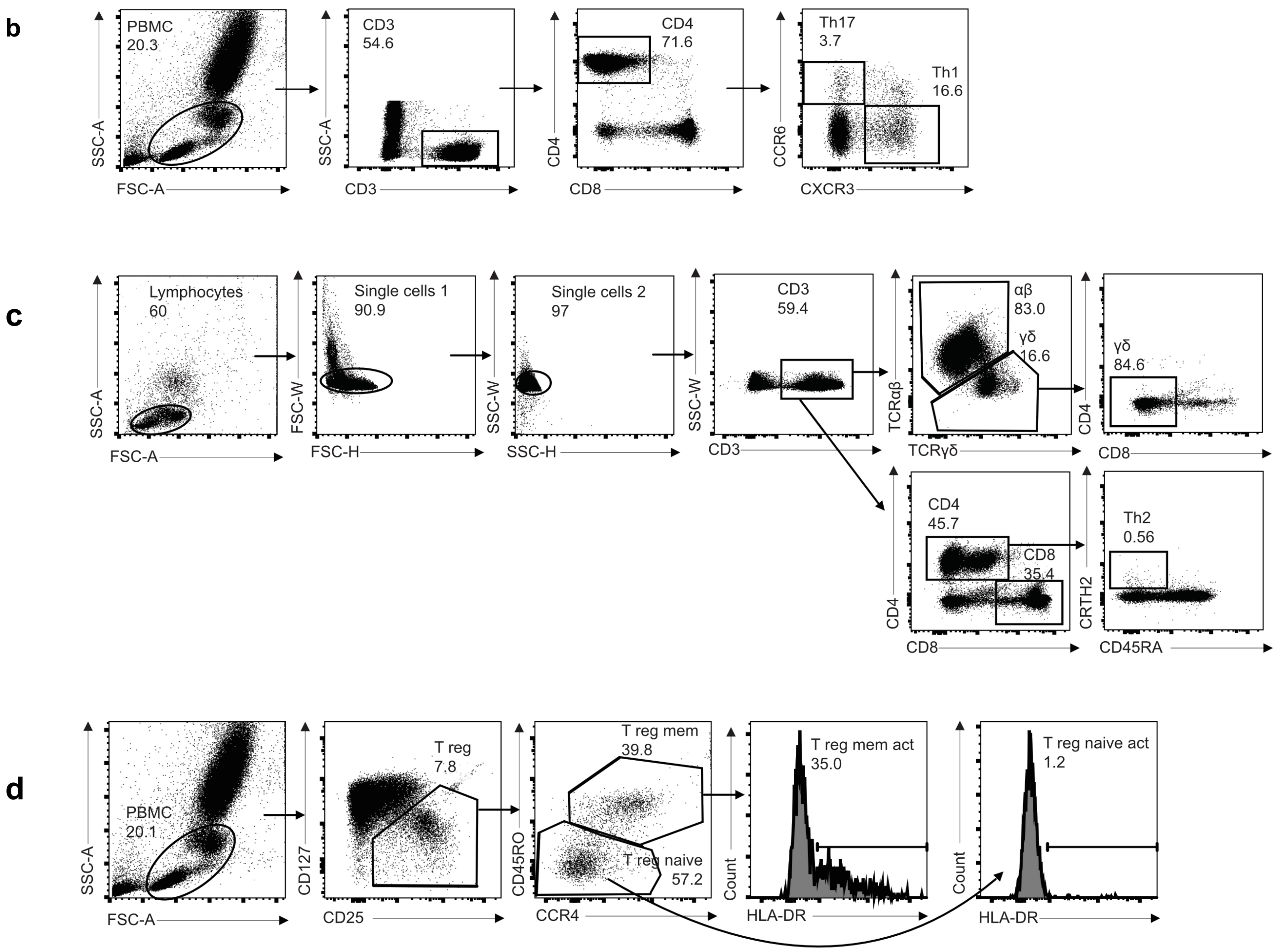

Fig. S1 Gating strategy used for flow cytometry

b T-helper subsets Th1 and Th17

c T-helper subset Th2 and γδ T lymphocytes

d Regulatory T lymphocytes

SSC-A, Side-scatter area; FSC-A, forward-scatter area; FSC-H, forward-scatter height.

e

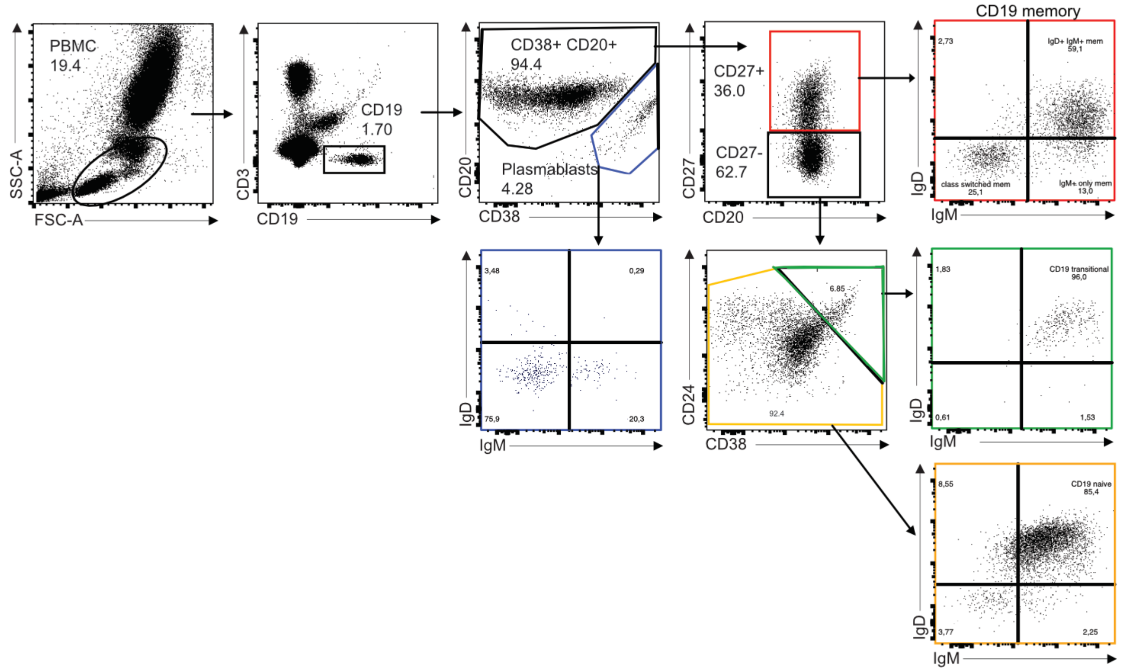

Fig. S1e Gating strategy used for flow cytometry, B lymphocytes

SSC-A, Side-scatter area; FSC-A, forward-scatter area; FSC-H, forward-scatter height.

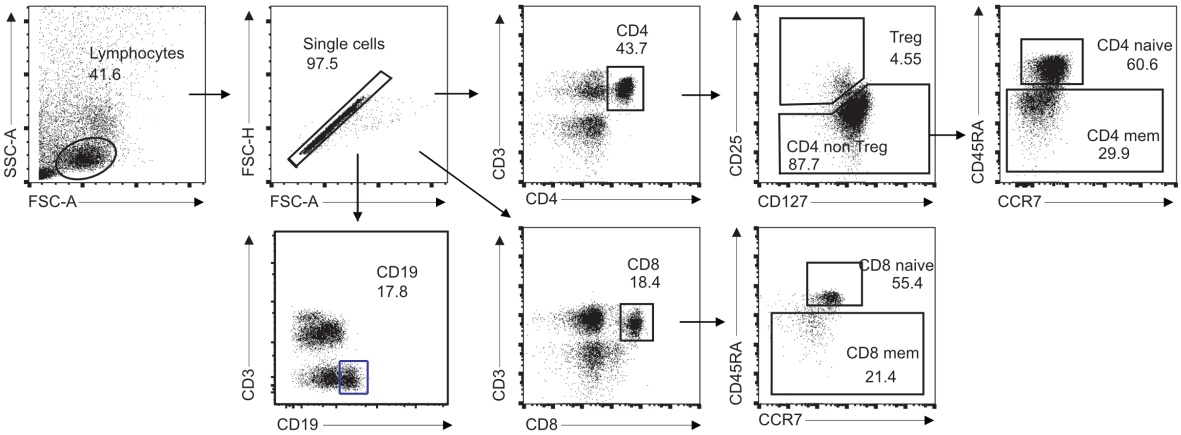

Fig. S2 Gating strategy for the cell sorting

B lymphocytes, naïve and memory T helper lymphocytes, and naïve and memory cytotoxic T lymphocytes were sorted.

SSC-A, Side scatter area; FSC-A, forward scatter area; FSC-H, forward scatter height.

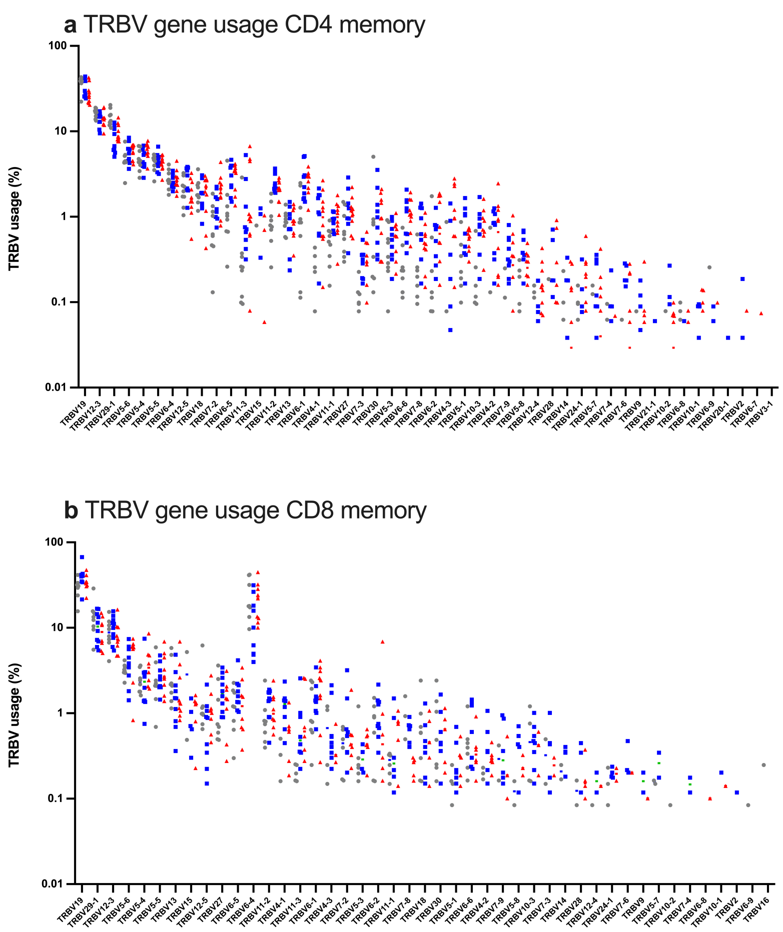

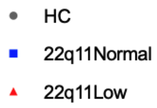

Fig. S3 TRBV and TRBJ gene usage

a Relative frequencies of TRBV gene usage in CD4 memory cells and **b** CD8 memory cells. **c** TRBJ gene usage in CD4 naïve, **d** CD4 memory, **e** CD8 naïve and **f** CD8 memory cells. Definitions of the cell types are provided in Supplementary Table S1. Results are shown as individual values for the healthy controls (gray dots), 22q11Normal patients (blue squares), and 22q11Low

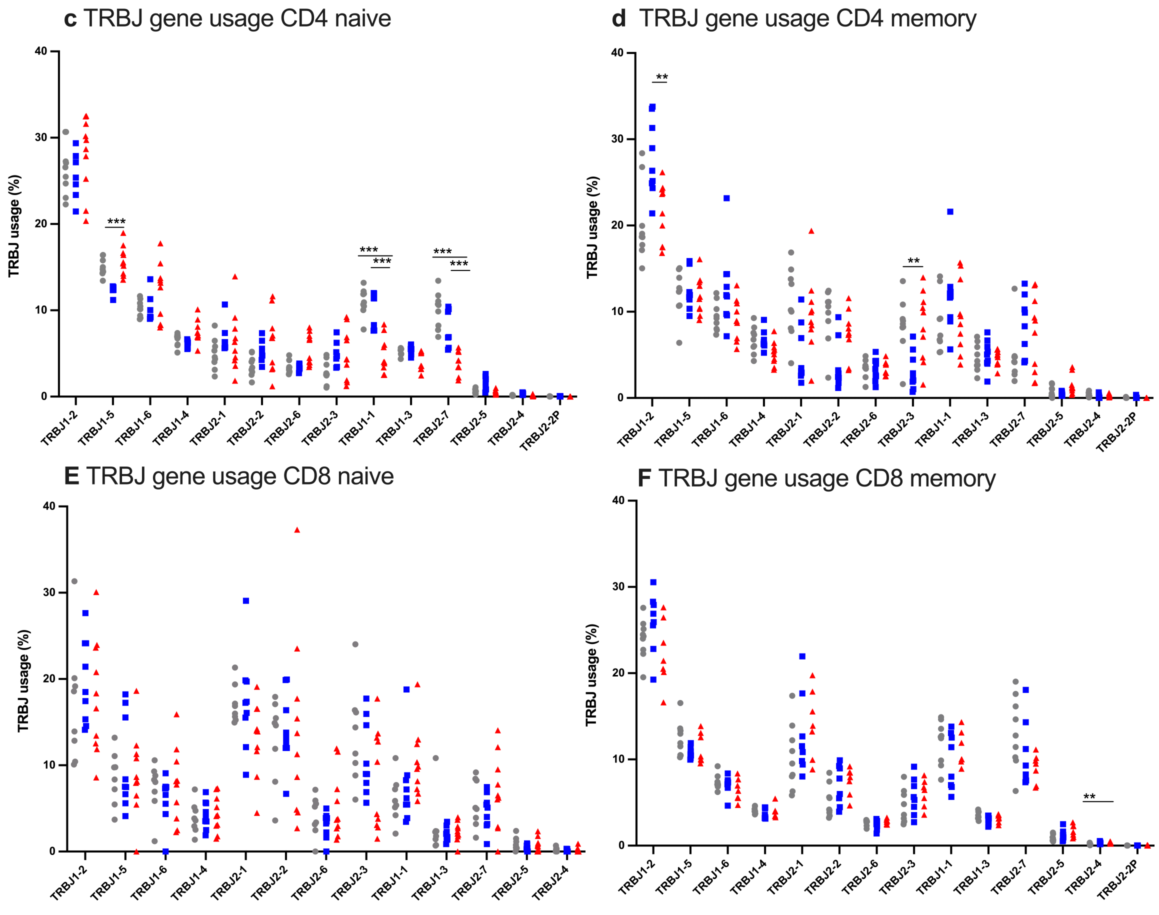

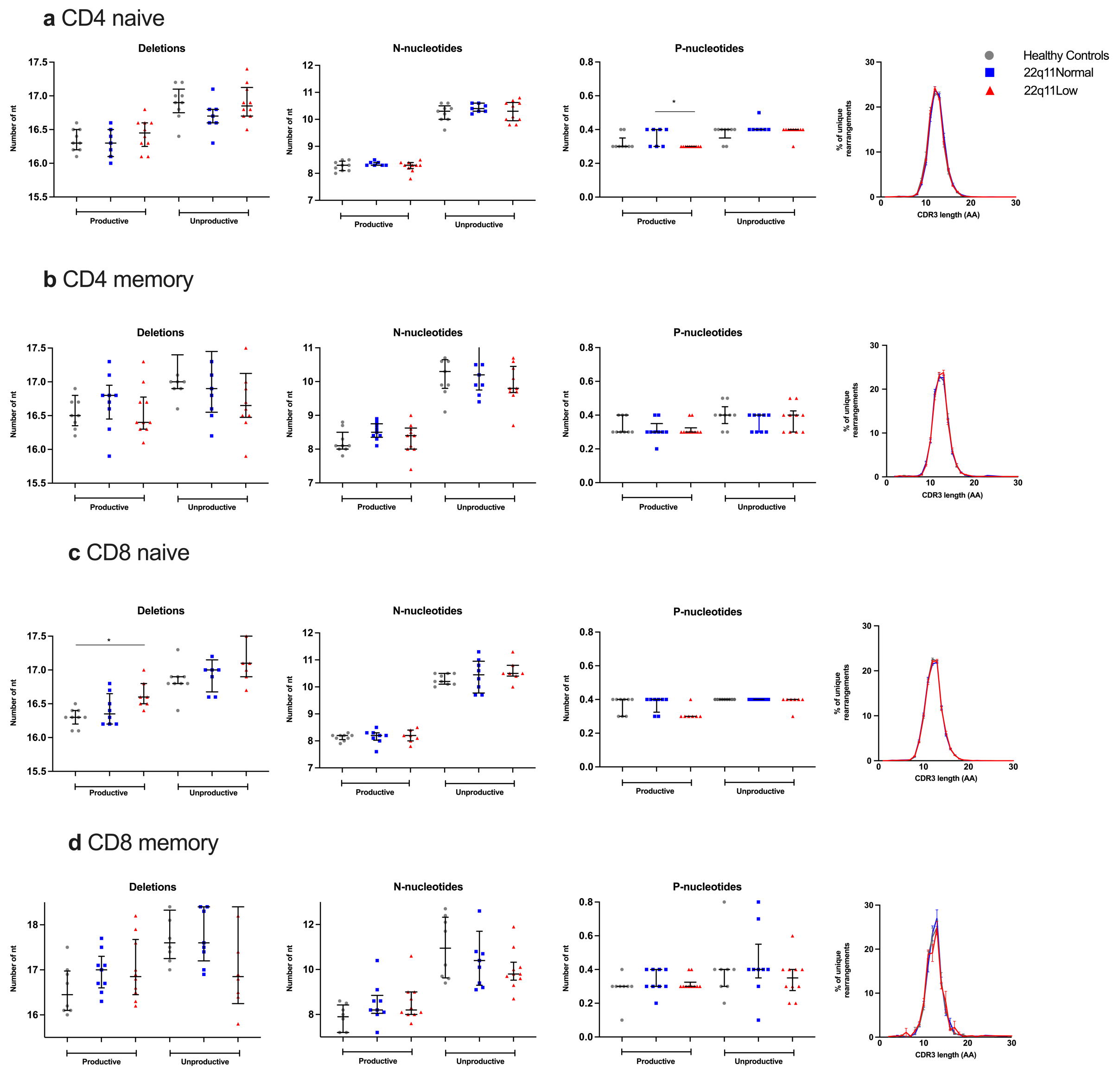

Fig. S4 TRB junction characteristics of the T-lymphocyte populations

a Junctional deletions and insertions of N- and P-nucleotides and CDR3 length distribution in the CD4 naïve, **b** CD4 memory, **c** CD8 naïve and **d** CD8 memory cells. Definitions of the cell types are provided in Supplementary Table S1.

Results are shown as individual values for the healthy controls (gray dots), 22q11Normal individuals (blue squares), and 22q11Low individuals (red triangles). Information on missing data is provided in Supplementary Table S3. Lines denote medians and whiskers indicate the interquartile ranges. Results in the CDR3 length distribution plots are presented as group mean values with the whiskers representing SEM. *P*-value summary indicated on each graph: **P* ≤ .05.
